# A conserved, N-terminal tyrosine signal directs Ras for inhibition by Rabex-5

**DOI:** 10.1371/journal.pgen.1008715

**Published:** 2020-06-19

**Authors:** Chalita Washington, Rachel Chernet, Rewatee H. Gokhale, Yesenia Martino-Cortez, Hsiu-Yu Liu, Ashley M. Rosenberg, Sivan Shahar, Cathie M. Pfleger

**Affiliations:** 1 Department of Oncological Sciences, The Icahn School of Medicine at Mount Sinai, New York, New York, United States of America; 2 University of Cincinnati College of Medicine, Cincinnati, Ohio, United States of America; 3 The Graduate School of Biomedical Sciences, The Icahn School of Medicine at Mount Sinai, New York, New York, United States of America; 4 The Tisch Cancer Institute, The Icahn School of Medicine at Mount Sinai, New York, New York, United States of America; 5 Tufts University School of Medicine, Boston, Massachusetts, United States of America; 6 Memorial Sloan Kettering Cancer Center, New York, New York, United States of America; 7 Columbia University, New York, New York, United States of America; 8 New York Medical College, Valhalla, New York, United States of America; Skirball Institute of Biomolecular Medicine - New York University Medical Center, UNITED STATES

## Abstract

Dysregulation of the Ras oncogene in development causes developmental disorders, “Rasopathies,” whereas mutational activation or amplification of Ras in differentiated tissues causes cancer. Rabex-5 (also called RabGEF1) inhibits Ras by promoting Ras mono- and di-ubiquitination. We report here that Rabex-5-mediated Ras ubiquitination requires Ras Tyrosine 4 (Y4), a site of known phosphorylation. Ras substitution mutants insensitive to Y4 phosphorylation did not undergo Rabex-5-mediated ubiquitination in cells and exhibited Ras gain-of-function phenotypes *in vivo*. Ras Y4 phosphomimic substitution increased Rabex-5-mediated ubiquitination in cells. Y4 phosphomimic substitution in oncogenic Ras blocked the morphological phenotypes associated with oncogenic Ras *in vivo* dependent on the presence of Rabex-5. We developed polyclonal antibodies raised against an N-terminal Ras peptide phosphorylated at Y4. These anti-phospho-Y4 antibodies showed dramatic recognition of recombinant wild-type Ras and Ras^G12V^ proteins when incubated with JAK2 or SRC kinases but not of Ras^Y4F^ or Ras^Y4F,G12V^ recombinant proteins suggesting that JAK2 and SRC could promote phosphorylation of Ras proteins at Y4 *in vitro*. Anti-phospho-Y4 antibodies also showed recognition of Ras^G12V^ protein, but not wild-type Ras, when incubated with EGFR. A role for JAK2, SRC, and EGFR (kinases with well-known roles to activate signaling through Ras), to promote Ras Y4 phosphorylation could represent a feedback mechanism to limit Ras activation and thus establish Ras homeostasis. Notably, rare variants of Ras at Y4 have been found in cerebellar glioblastomas. Therefore, our work identifies a physiologically relevant Ras ubiquitination signal and highlights a requirement for Y4 for Ras inhibition by Rabex-5 to maintain Ras pathway homeostasis and to prevent tissue transformation.

## Author summary

Ras and Ras-related genes play important roles in how an organism develops in addition to maintaining health as an adult. Failure to properly regulate Ras proteins can lead to a range of diseases including cancer. Our study identifies an important aspect of how Ras is recognized by one of its inhibitors. This knowledge could be a first step in developing new anti-Ras therapeutics for Ras-associated cancers.

## Introduction

In *Drosophila*, Ras proteins are represented by Ras1/Ras85D and Ras2/Ras64B. *Drosophila* Ras1/Ras85D corresponds to mammalian H-Ras, N-Ras, and K-Ras and is distinct from the closely related Ras2/Ras64B protein represented by R-Ras in mammals. Using the convention that predominates in the literature, here we refer to *Drosophila* Ras1/Ras85D as Ras; we refer collectively to mammalian H-Ras, N-Ras, and K-Ras as Ras or individually to specific isoforms as H-Ras, N-Ras, and K-Ras as appropriate; and we refer to Ras2/Ras64B as Ras2 for the *Drosophila* protein and R-Ras for the mammalian protein. *Drosophila* Ras and mammalian H-Ras N-Ras and K-Ras share sequence identity in their N-termini but diverge in a C-terminal region called the HyperVariable Region or HVR [1; for review, 2–3] (depicted schematically in S1 Fig). It is well accepted that one of the roles of the HVR is to direct the membrane association of Ras in part by the C-terminal CAAX box (cysteine, aliphatic amino acid, aliphatic amino acid, any amino acid) [1; for review, 2–3].

Ras signaling regulates cell proliferation, growth, differentiation, and cell survival by signaling to a range of downstream effectors including Raf/ERK/MAPK, RalGDS, and PI3K among others [1–12]. Consequently, Ras dysregulation in development alters patterning and causes developmental disorders collectively called “Rasopathies” [for review, 4–9]. Mutational activation and amplification of Ras in differentiated tissues are implicated in cancer [for review, 10–12]. Therefore, mechanisms of attenuating Ras activity are crucial for proper development and to prevent disease.

Rabex-5 (also called RabGEF1), an A20-like E3 ubiquitin ligase, promotes inhibitory mono- and di-ubiquitination of *Drosophila* Ras and mammalian H-Ras and N-Ras to restrict signaling to downstream effectors [13–16]. Rabex-5 inhibits both wild-type Ras and also the constitutively active oncogenic mutant Ras^G12V^ (also referred to as RasV12 in the literature) [13–16].

No signal in Ras has been reported to direct its inhibition by Rabex-5, and no ubiquitination targeting motif has been ascribed to Rabex-5 or the A20 family of ubiquitin ligases. We mapped a ubiquitination signal in *Drosophila* Ras; we report here that Rabex-5 inhibition of *Drosophila* Ras requires Ras N-terminal tyrosine 4 (Y4). Phenylalanine substitution mutants of Ras at Y4 (to prevent phosphorylation) were insensitive to Rabex-5-mediated ubiquitination in S2 cells and showed Ras gain-of-function phenotypes *in vivo*. Glutamic acid substitution mutants of Ras at Y4 (to mimic the charge of phosphorylation) showed increased Rabex-5-mediated ubiquitination in S2 cells, and glutamic acid substitution mutants of Ras^G12V^ at Y4 suppressed oncogenic Ras phenotypes *in vivo*, dependent on the presence of Rabex-5. JAK2 and SRC kinases are capable of promoting phosphorylation of Ras^WT^ at Y4, whereas JAK2, SRC, and EGFR can promote phosphorylation of an oncogenic form of Ras, Ras^G12V^, at Y4 as measured by recognition by anti-pY4 antibodies.

## Results and discussion

### An N-terminal tyrosine-based signal directs Ras for mono- and di-ubiquitination

To elucidate the molecular mechanism of Ras inhibition by Rabex-5 and to advance our understanding of the A20 family of E3 ubiquitin ligases, we mapped a signal in *Drosophila* Ras responsible for Rabex-5 mediated ubiquitination with a deletion strategy (S1A Fig). In our previous work, we used a double FLAG-His6 tag on full length Ras [13, 16]. Deletion constructs were tagged with a triple tag of GFP-FLAG-His6 so that smaller constructs (corresponding to larger deletions) would be large enough to eliminate concerns of peptide instability. Ubiquitin conjugates of deletion constructs were isolated from Schneider S2 cells using nickel purification (to isolate the His6 tag). Visualization of the conjugates was achieved using the FLAG tag (to visualize Ras) and HA (to visualize ubiquitin which was expressed from an HA-Ub plasmid) as done previously [13, 16]. Ras is also regulated by the E3s Nedd4 [17], βTRCP [18], and LZTR1 [19–20]. Therefore, to map a Rabex-5 ubiquitination signal but without excluding Nedd4, βTRCP, or LZTR1 signals, our initial deletion strategy followed ubiquitination of Ras in Schneider S2 cells without Rabex-5 supplementation (S1A–S1D Fig). As noted, Ras membrane association is directed by a C-terminal CAAX signal which is represented by the amino acids CKML in *Drosophila* Ras. To properly localize N-terminal constructs, we tagged each deletion construct at its C-terminus with the *Drosophila* Ras CAAX box CKML (depicted schematically in S1A Fig; sequences listed in the methods section).

**Fig 1:**
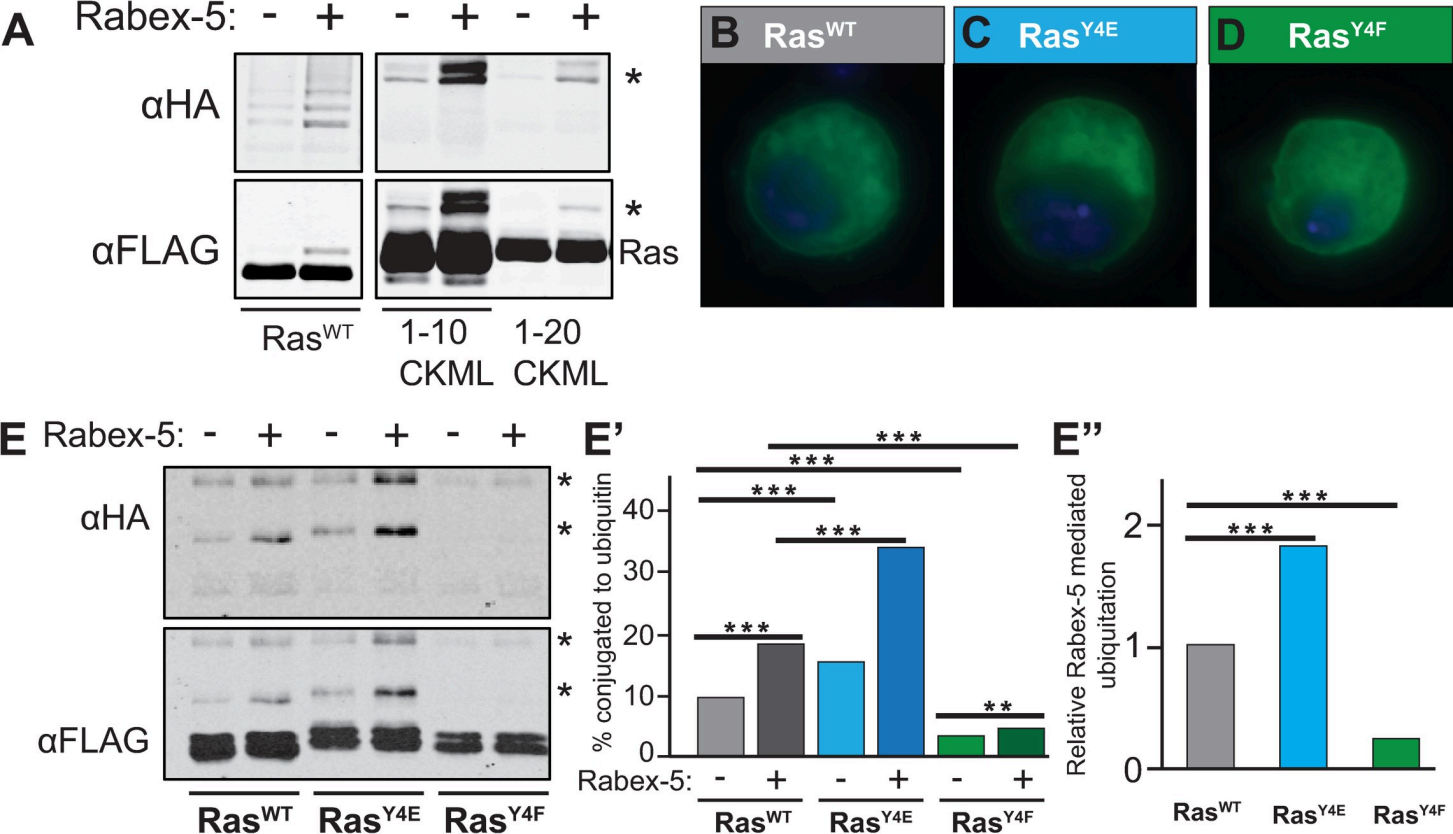
Ras Tyrosine 4 is required for Rabex-5-mediated Ras ubiquitination. (A) Flag-His6-GFP tagged Ras (Ras^WT^) or FLAG-His6-GFP tagged Ras N-terminal fragments tagged with C-terminal localization signal CKML (1–10 CKML, 1–20 CKML) were co-transfected into Schneider S2 cells with HA-Ub with or without Rabex-5 and purified on nickel beads as done previously [13, 16]. The N-terminal 10 amino acids of Ras contain a signal sufficient to confer ubiquitination onto GFP in the pattern of full length Ras and to support Rabex-5-mediated ubiquitination (image of entire gel in S1G Fig). The bands recognized by both anti-FLAG (the tag on Ras) and anti-HA (the tag on ubiquitin) antibodies represent ubiquitinated species of Ras and are marked by an asterisk, *. Other bands in the anti-HA gel reflect non-Ras, co-purifying ubiquitinated proteins. (B-D) Localization in S2 cells of FLAG-His6 tagged Ras^WT^ (B), Ras^Y4E^ (C), and Ras^Y4F^ (D) visualized by staining for FLAG. Boxes represent 20 μm square regions. (E) Western blot of FLAG-His6 Ras^WT^ and Y4 mutants purified from S2 cells on nickel beads. Ubiquitin conjugates (anti-HA antibodies, upper blot) and total Ras (anti-FLAG antibodies, lower blot) show an increase in basal ubiquitination for phoshomimic Ras, Ras^Y4E^, compared to Ras^WT^ (lane 3 compared to lane 1) and Rabex-5-mediated ubiquitination (increase in lane 4 compared to lane 3 versus the increase in lane 2 compared to lane 1). Non-phosphorylatable Ras, Ras^Y4F^, shows decreased basal ubiquitination (lane 5 compared to lane 1) and less responsiveness to Rabex-5 (lane 6 compared to lane 5). Quantification of these experiments shows the percent of Ras conjugated to ubiquitin (graph in E’) and the relative Rabex-5 mediated ubiquitination (graph in E”). *** indicates p<0.0001 from CHITEST function in Excel for Chi-square statistical analysis comparing the percentage of Ras construct in unconjugated or ubiquitin conjugated species between indicated samples. Western Analysis utilized the Licor Odyssey. Gels in this figure showed non-saturating band intensities; quantification of bands in E’ and E” utilized non-saturating signals in the linear range.

Previous work by Jura et al. reported the importance of the HVR for Ras ubiquitination. Inhibitory ubiquitination of H-Ras and N-Ras but not K-Ras was reported in mammalian cells [15]. Notably, replacing the K-Ras HVR with the H-Ras HVR conferred ubiquitination onto K-Ras [15]. This could have reflected the requirement for specific sequences in the H-Ras HVR not present in the K-Ras HVR; alternatively, this could have reflected the importance of the HVR in directing the localization of each Ras isoform to a compartment where the ubiquitination occurs. We report here that the *Drosophila* Ras HVR was neither sufficient nor required for Ras ubiquitination. C-terminal constructs were not ubiquitinated (S1A and S1B Fig). After narrowing the region sufficient for ubiquitination to the N-terminal 20 amino acids of Ras, we tested the ability of the N-terminal 20 amino acids to serve as a competitive inhibitor. Expressing GFP-Myc-tagged 1-20CKML peptides in excess prevented the formation of Ras-ubiquitin conjugates of full-length FLAG-His6 tagged Ras^WT^ isolated on nickel beads and detected by anti-HA antibodies (S1E Fig), whereas GFP-myc tagged peptides of a different 20 amino acid region in excess had no effect on Ras^WT^ ubiquitin conjugates (S1E Fig). We further narrowed the region sufficient to confer Ras ubiquitination in S2 cells to the N-terminal 10 amino acids of Ras (Fig 1A, S1A Fig, S1F and S1G Fig). Co-transfecting cells with Rabex-5 increased the ubiquitination of this region (Fig 1A, S1F and S1G Fig) but not of other small regions of Ras (S1F Fig).

Because the HVR was neither sufficient for ubiquitination nor required for ubiquitination of constructs, our results taken together with the previous work of Jura et al.[15], could be consistent with a requirement for the HVR to direct Ras localization [1; for review, 2–3] to a specific compartment where a sequence shared by H-Ras and K-Ras would then be recognized. In fact, the N-termini of *Drosophila* Ras and H-Ras, N-Ras, and K-Ras are identical (alignment, S2C Fig). While we cannot rule out a contribution from amino acids 11–189, the first 10 amino acids of Ras were sufficient to confer ubiquitination.

Normally, Ras^WT^ and Ras^G12V^ proteins associate with the plasma membrane and are also found in the cytoplasm [1–3, 15] (Fig 1B, S1H Fig S2E–S2E” Fig). Previous work by the Bar-Sagi group has shown that a non-ubiquitinated mutant of mammalian H-Ras (in which all solvent exposed lysines were mutated to arginine) had increased localization to the Golgi from which Ras proteins are known to actively signal [15]. In contrast, an H-Ras ubiquitin fusion protein showed decreased Golgi localization and increased localization in an early endosomal compartment [15]. This suggests that the mechanism of inhibitory ubiquitination is to sequester ubiquitinated Ras proteins away from downstream effectors they would encounter at the membrane or in the Golgi by retaining it in the early endosome. We over-expressed Rabex-5^DPYT^ (which maintains E3 activity but has impaired Rab5 GEF function) to increase the inhibitory ubiquitination of endogenous Ras proteins without affecting the endosomal compartment in the developing eye using *GMR-gal4*. Staining for endogenous Ras using anti-pan-Ras antibodies at a specific time window 48 hours after puparium formation revealed a dramatic redistribution of Ras to an intracellular compartment (S1I Fig) not seen in controls (S1H Fig). Taken together with work from the Bar-Sagi group showing redistribution of a Ras ubiquitin fusion construct [15], we speculate that Rabex-5 activity can promote Ras re-localization to prevent its signaling to downstream effectors in a highly conserved fashion.

Most cellular Ras is GDP-loaded [21]. Mutation at or close to Ras codon 12 biases Ras to a GTP-loaded conformation [22]. To define a signal in both GDP- and GTP-loaded Ras conformations, we created alanine substitution mutants of the first ten amino acids in FLAG-His6 tagged full-length *Drosophila* Ras^WT^ and FLAG-His6 tagged full-length *Drosophila* Ras^G12V^. Y4A and V7A substitution reproducibly decreased ubiquitination in both Ras^WT^ and Ras^G12V^ (S2A and S2B Fig). Curiously, we saw differences in alanine scanning between Ras and Ras^G12V^ constructs (S2A and S2A’ Fig, summarized in S2B Fig). E3A and K5A substitutions decreased ubiquitination of only Ras^G12V^ (S2A’ Fig, S2B Fig).

The tyrosine and valine important for ubiquitination of *Drosophila* Ras^WT^ and Ras^G12V^ and the lysine important for ubiquitination in Ras^G12V^ are entirely conserved in human H-Ras, N-Ras, and K-Ras as well as in *Drosophila Ras2* (S2C Fig). To establish if Rabex-5 can also promote ubiquitination of Ras2, we expressed FLAG-His6 tagged Ras2 in Schneider S2 cells. Rabex-5 promoted Ras2 ubiquitination (S2D Fig) to a similar extent as Ras^WT^. The crucial tyrosine is not conserved in R-Ras (alignment, S2C Fig), so it is unclear if the ability of Rabex-5 to promote Ras2 ubiquitination would be conserved for human R-Ras.

In transfected Schneider S2 cells, most cells show Ras associated with the membrane and also in intracellular puncta in the cytoplasm. In a population of transfected cells, some individual cells showed enrichment of Ras signal at the membrane or in intracellular puncta (S2E–S2E” Fig). Because work by the Bar-Sagi group indicated the importance of localization for Ras ubiquitination [15], we assessed localization of the alanine scanning constructs. Localization of each alanine mutant disrupting ubiquitination confirmed that a lack of ubiquitination did not result from a failure to localize to the plasma membrane and these cytoplasmic puncta (S2F–S2M Fig).

Previous proteomic studies have identified phosphorylation in mammalian Ras at Y4 [23] and also at other tyrosines [24–30]. Ras^WT^ purified from S2 cells was recognized by anti-phosphotyrosine (p-tyr) antibodies (S2N–S2P Fig). Ras^Y4F^ (phenylalanine substitution to preserve structure but without the hydroxyl group) showed decreased recognition by anti-p-tyr antibodies compared to Ras^WT^ (S2O and S2P Fig) consistent with phosphorylation at Y4 and also at other tyrosines in *Drosophila* Ras as seen in mammalian Ras.

### Negative charge at Y4 increases Rabex-5 mediated Ras ubiquitination

We created glutamic acid substitution mutant Ras^Y4E^ to mimic the charge of phosphorylation in order to test the effects of negative charge at Y4 *in vitro* and *in vivo*. Ras^Y4E^ and Ras^Y4F^ associated with the plasma membrane and intracellular puncta as seen with Ras^WT^ (Fig 1B–1D). The basal level of Ras^Y4E^ ubiquitination increased compared to Ras^WT^, and Rabex-5-mediated Ras^Y4E^ ubiquitination increased compared to Rabex-5-mediated ubiquitination of Ras^WT^ (Fig 1E, quantified in Fig 1E’ and 1E”, raw data in S1 File). In contrast, Ras^Y4F^ showed lower basal ubiquitination and no Rabex-5-mediated ubiquitination (Fig 1E–1E”).

### Ras^Y4F^ shows Ras gain-of-function phenotypes *in vivo*

To investigate a biological role for *Drosophila* Ras Y4, we created inducible transgenic lines for Ras^WT^, Ras^Y4E^, Ras^Y4F^, Ras^G12V^, and double mutants Ras^Y4E,G12V^ and Ras^Y4F,G12V^. Each transgene was tagged with the N-terminal FLAG-His6 tag used in our *in vitro* studies and was inserted at the same attp40 genomic site to rule out position insertion effects. Transgenes expressed at similar levels (S3B Fig for larvae). Ras^Y4E^ and Ras^Y4F^ expressed with *Act5C-gal4* rescued the early lethality of Ras loss-of-function alleles to the same extent as Ras^WT^ transgenes (S3I Fig), suggesting that genetically Y4E and Y4F mutations are capable of carrying out Ras function and do not inactivate Ras inherently.

**Fig 2:**
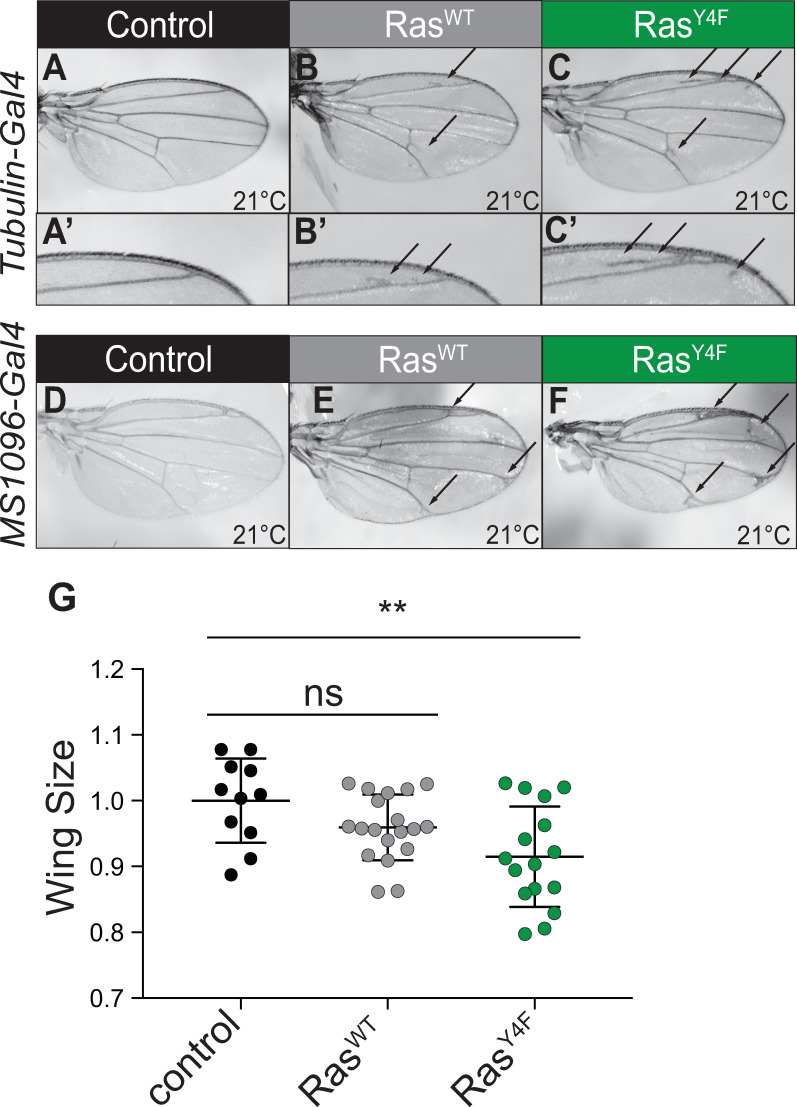
Non-phosphorylatable Ras shows Ras gain-of-function phenotypes *in vivo*. (A) Control wing (*Tub-gal4/+*). (B) Ras^WT^ expression driven by *Tub-gal4*. Ectopic longitudinal vein material is seen anterior to the L2 longitudinal vein (arrow, enlarged in B’) and on the posterior crossvein (arrow). (C) Ras^Y4F^ expression driven by *Tub-gal4*. Ectopic longitudinal vein material is seen anterior and posterior to the L2 longitudinal vein (arrows, enlarged in C’). The ectopic wing vein phenotype is enhanced upon Y4F mutation (compare C’ to B’) (arrow). (D) Control homozygous *MS1096-gal4* wing. (E) Wing homozygous for *MS1096-gal4* and *UAS Ras*^*WT*^. Extra wing vein material is obvious, particularly where the longitudinal veins meet the wing margin (arrows). (F) Wing homozygous for *MS1096-gal4* and *UAS Ras*^*Y4F*^. The extra wing vein phenotype (arrows) is enhanced compared to Ras^WT^. (G) Quantification of wing area for wings shown in D-F. “ns” = not significant. ** = p<0.005. Female wings are shown. For male wings, see S3 Fig. Genotypes for wings in this and subsequent figures are indicated in the Methods section.

When Ras signaling is kept within an appropriate range during development, growth, proliferation, and cell fate decisions occur normally. When Ras signaling is in excess, a variety of growth and patterning phenotypes result depending on the developmental timing and tissue context. Expression of Ras^WT^ in many contexts such as in the eye (using *GMR-gal4* and *ey-gal4*) and the wing (using *c765-gal4*) does not disrupt growth or disrupt patterning and cell fate decisions (summarized in S3A Fig), presumably because endogenous mechanisms of regulating Ras buffer the increased Ras expression to within the normal range. However, expressing Ras^WT^ with constitutive driver *Tub-gal4* or dorsal wing driver *MS1096-gal4* resulted in the Ras gain-of-function phenotype of ectopic wing veins (Fig 2B, 2B’ and 2E; S3D and S3D’ Fig, S3G Fig) compared to control wings (Fig 2A, 2A’ and 2D; S3C and S3C’ Fig, S3F Fig). Ras^Y4F^ increased wing vein disruption (Fig 2C, 2C’ and 2F; S3E and S3E’ Fig, S3H Fig) compared to Ras^WT^ and statistically significantly decreased wing size compared to controls (Fig 2G, raw data in S2 File). The increase in severity of phenotype suggests that Y4F substitution increases Ras activity.

### Y4F substitution enhances oncogenic Ras phenotypes *in vivo*

**Fig 3:**
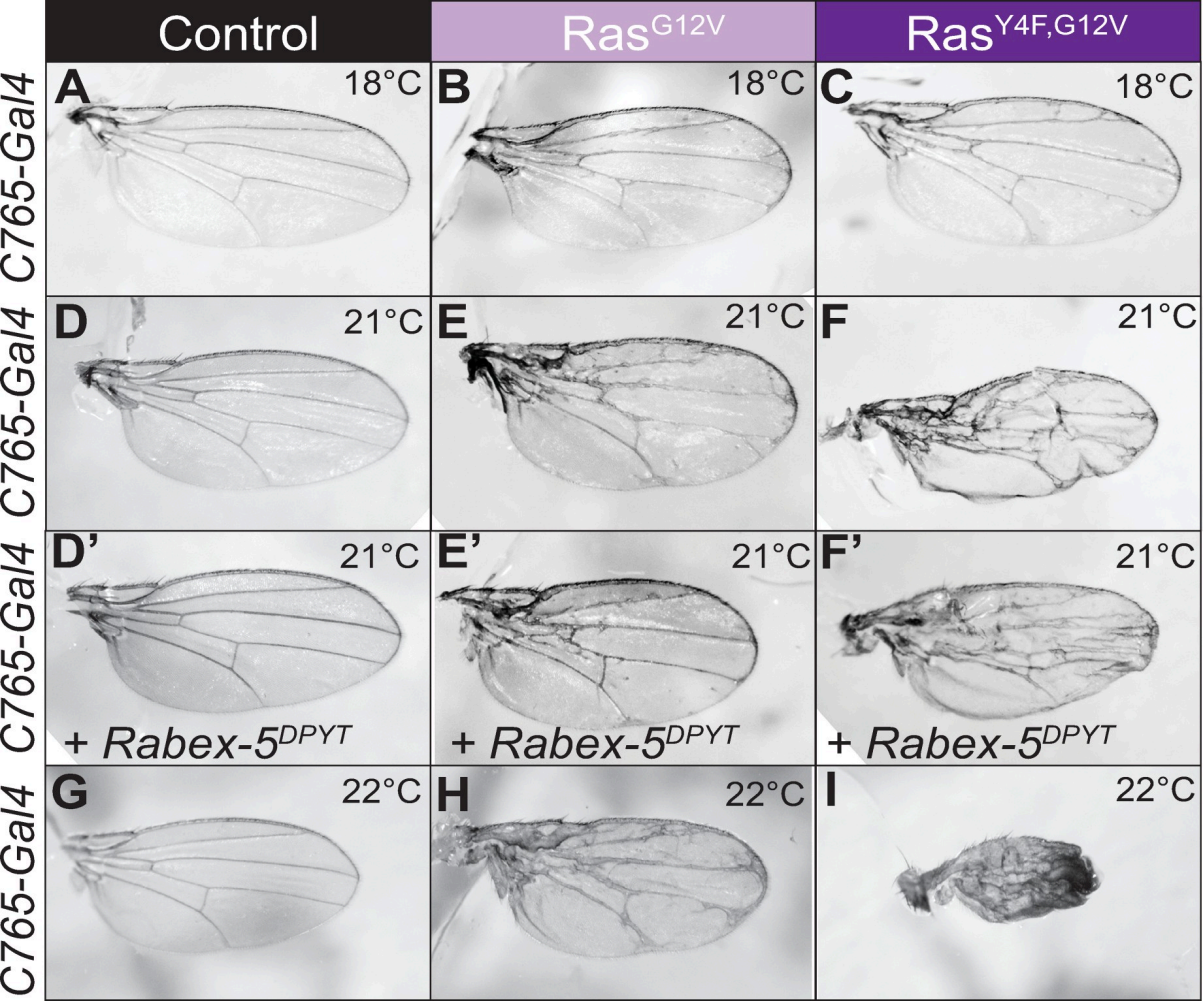
Non-phosphorylatable oncogenic Ras enhances oncogenic Ras phenotypes. Increasing temperature increases Gal4/UAS-mediated expression of transgenes. (A) Control wing (*c765-gal4/+*) at 18°C. (B) Oncogenic Ras, Ras^G12V^, expressed using *c765-gal4* at 18°C causes subtle vein abnormalities. (C) Y4F mutation in oncogenic Ras, Ras^Y4F,G12V^, shows an increase in wing vein effects. (D) Control wing (*c765-gal4/+*) at 21°C. (D’) Control wing expressing low level of Rabex-5^DPYT^ using *c765-gal4* at 21°C. This level of expression is not sufficient to disrupt wing vein pattern. (E) Ras^G12V^, expressed using *c765-gal4* at 21°C, causes extra wing veins and thickened veins. (E’) Rabex-5^DPYT^ expression concurrent to Ras^G12V^, using *c765-gal4* at 21°C suppresses the extra wing veins and thickened vein phenotypes. (F) Ras^Y4F,G12V^ expressed using *c765-gal4* at 21°C shows an increase in wing effects including reduction in size compared to Ras^G12V^. (F’) Rabex-5^DPYT^ expression concurrent to Ras^Y4F,G12V^ using *c765-gal4* at 21°C shows a similar phenotype as Ras^Y4F,G12V^. (G) Control wing (*c765-gal4/+*) at 22°C. (H) Ras^G12V^, expressed using *c765gal4* at 22°C causes a more severe phenotype than at 21°C. (I) Ras^Y4F,G12V^ expressed using *c765-gal4* at 22°C shows further wing disruption compared to Ras^G12V^. Female wings are shown; for male wings, see S4 Fig.

Expressing Ras^G12V^ in the wing causes wing vein abnormalities (Fig 3B, 3E and 3H; S4B Fig, S4E Fig, S4H Fig) compared to controls (Fig 3A, 3D and 3G; S4A Fig, S4D Fig, S4G Fig). Y4F mutation in oncogenic Ras, Ras^Y4F,G12V^, enhanced wing vein abnormalities and further reduced wing size and caused some wings to appear crumpled (Fig 3C, 3F and 3I; S4C Fig, S4F Fig, S4I Fig). Increasing Rabex-5 E3 activity by co-expressing Rabex-5^DPYT^ [13, 31] to a level with no wing phenotype (Fig 3D’; S4D’ Fig) suppressed Ras^G12V^ phenotypes (Fig 3E’; S4E’ Fig) but did not suppress Ras^Y4F,G12V^ phenotypes (Fig 3F’; S4F’ Fig). The enhanced phenotypes of Ras^Y4F,G12V^ compared to Ras^G12V^ and the suppression of Ras^G12V^ but not Ras^Y4F,G12V^ by Rabex-5 E3 activity are consistent with a model that Y4F mutation allows Ras^G12V^ to evade Rabex-5-mediated inhibition and emphasize the importance of Ras Y4 for targeting Ras for inhibition.

### Negative charge at Y4 suppresses oncogenic Ras phenotypes

**Fig 4:**
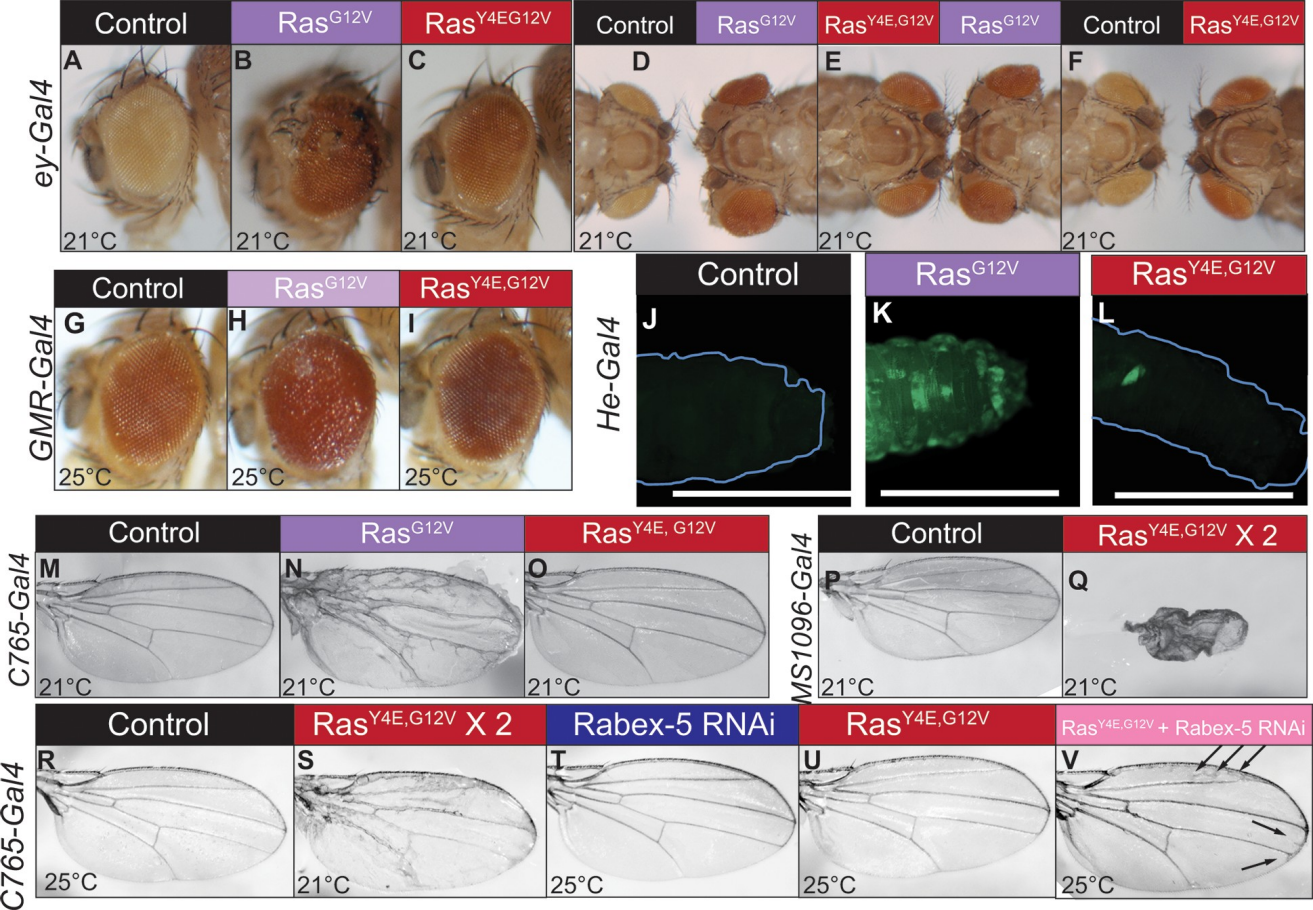
Ras Y4 phosphomimic suppresses the phenotypes of oncogenic Ras dependent on the presence of Rabex-5. (A-F) Y4E phosphomimic mutation suppresses the eye overgrowth and outgrowth phenotypes of Ras^G12V^. Control eye (*ey-gal4/+*) (A, left eye in D, left eye in F). Ras^G12V^, driven by *ey-gal4* (B, right eye in D and E). Ras^Y4E,G12V^ driven by *ey-gal4* (C, left eye in E, right eye in F). Head-to-head photos in D-F highlight the suppression of overgrowth. (G) Control *GMR-gal4/+* eye. (H) Ras^G12V^ driven by *GMR-gal4*. (I) Ras^Y4E,G12V^ driven by *GMR-gal4*. Y4E phosphomimic mutation suppresses the rough eye and black tissue phenotypes of Ras^G12V^. Female eyes are shown in A-I. For male eyes, see S5A–S5J Fig. (J-L) *He-gal4* was used to drive Ras transgene expression in hemocytes. To visualize hemocytes, a *UAS GFP* transgene was also used. (J) Control, GFP driven by *He-gal4*. (K). Ras^G12V^ and GFP driven by *He-gal4*. (L). Ras^Y4E,G12V^ and GFP driven by *He-gal4*. Larvae in J-L were imaged at the same settings. Tracings of larvae in J and L indicate larval outlines. Excess hemocytes are evident in (K) by the strong GFP signal (green). The excess hemocyte phenotype is suppressed upon Y4E mutation. Scale bars in J-L indicate 1.5 mm. Images of the entire larvae are shown in S5K–S5M Fig. (M) Control wing *(c765-gal4/+)*. (N) Ras^G12V^ driven by *c765-gal4*. (O) Ras^Y4E,G12V^ driven by *c765-gal4*. Y4E phosphomimic mutation suppresses the extra wing vein phenotype of Ras^G12V^. (P) Control homozygous *MS1096-gal4* wing. (Q) Wing homozygous for *MS1096-gal4* and Ras^Y4E,G12V^. One copy of oncogenic Ras driven by *ms1096gal4* is lethal (therefore wings cannot be shown); Y4E phosphomimic mutation yields obvious wing phenotypes but suppresses the lethality of expressing two copies of Ras^G12V^. (R) Control *c765-gal4/+* wing. (S) Wing homozygous for *c765-gal4* and Ras^Y4E,G12V^ transgene show the obvious extra wing vein phenotype associated with oncogenic Ras. (T) Low-level Rabex-5 RNAi driven by *c765-gal4* yields no visible phenotype. (U) Ras^Y4E,G12V^ expression driven by *c765-gal4* shows very subtle or no extra wing vein phenotypes. (V) Ras^Y4E,G12V^ expression elicits obvious extra wing vein phenotypes (arrows) upon concurrent low-level Rabex-5 RNAi driven by *c765-gal4*. Female wings are shown in M-V; for male wings, see S5N–S5W Fig.

If preventing phosphorylation at Y4 allowed Ras to evade inhibition by Rabex-5, then mimicking Y4 phosphorylation would be predicted to increase targeting by Rabex-5, thus inhibiting Ras activity. Notably, Ras^Y4E,G12V^ exhibited suppressed phenotypes compared to Ras^G12V^ in all contexts tested. Ras^Y4E,G12V^ expression in the early eye with *ey-gal4* (Fig 4C, 4E and 4F; S5C Fig, S5E and S5F Fig) resembled a control eye (Fig 4A, 4D and 4F; S5A Fig, S5D Fig, S5F Fig) lacking the Ras^G12V^ overgrowth and tissue outgrowths (Fig 4B, 4D and 4E; S5B Fig, S5D and S5E Fig). Ras^Y4E,G12V^ expression later in eye development with *GMR-gal4* (Fig 4I, S5I and S5J Fig) resembled a control eye (Fig 4G, S5G Fig), not a rough Ras^G12V^ eye (Fig 4H, S5H–S5J Fig). Ras^Y4E,G12V^ expressed in hemocytes with *He-gal4* (Fig 4L, S5M Fig) resembled controls (Fig 4J, S5K Fig) not Ras^G12V^-induced hemocyte over-proliferation (Fig 4K, S5L Fig). Ras^Y4E,G12V^ expressed in the wing with *c765-gal4* (Fig 4O; S5P Fig) resembled a control wing (Fig 4M; S5N Fig) not the Ras^G12V^ wing vein phenotype (Fig 4N; S5O Fig). Ras^Y4E,G12V^ expressed in the dorsal wing with *MS1096-gal4* (Fig 4Q; S5R Fig) suppressed the lethality of Ras^G12V^ and caused disrupted wings compared to control wings (Fig 4P; S5Q Fig). These results demonstrate that negative charge at Ras Y4 is a mechanism for inhibiting Ras *in vivo*; this inhibition is strong enough to block oncogenic Ras visible phenotypes and to prevent oncogenic Ras^G12V^–induced lethality.

Ras^Y4E^ expressed with *Act5C-gal4* rescued the early lethality of Ras loss-of-function alleles to the same extent as Ras^WT^ transgenes (S3I Fig), and Ras^Y4E,G12V^ expressed in the dorsal wing promoted Ras phenotypes (Fig 4Q; S5R Fig). These data suggest that Y4E mutation does not inherently inactivate Ras, for example by causing misfolding. The ability of negative charge at Y4 to potently block oncogenic Ras activity is consistent with a model that Y4 phosphorylation leads to Ras inhibition by Rabex-5.

### Suppression of oncogenic Ras phenotypes by Y4 phosphomimic substitution requires Rabex-5

If Y4E substitution increased Ras targeting by Rabex-5 *in vivo* as seen *in vitro* (Fig 1E–1E”), then reducing Rabex-5 gene dosage should elicit oncogenic Ras phenotypes. Increased expression of Ras^Y4E,G12V^ resulted in obvious oncogenic Ras phenotypes (Fig 4S; S5T Fig) presumably due to overwhelming endogenous Rabex-5. Low-level Rabex-5 RNAi that on its own results in no obvious phenotype (Fig 4T; S5U Fig) and resembles a control wing (Fig 4R; S5S Fig) together with Ras^Y4E,G12V^ expression resulted in obvious ectopic wing veins (Fig 4V; S5W Fig). The lack of phenotype upon Ras^Y4E,G12V^ expression (Fig 4O and 4U; S5P Fig, S5V Fig) but extra wing vein phenotype upon concurrent Rabex-5 reduction (Fig 4V; S5W Fig) is consistent with a model that endogenous Rabex-5 strongly restricts Ras^Y4E,G12V^ activity.

### JAK2 and SRC kinases can promote phosphorylation of recombinant Ras^WT^ and Ras^G12V^ at Y4

**Fig 5:**
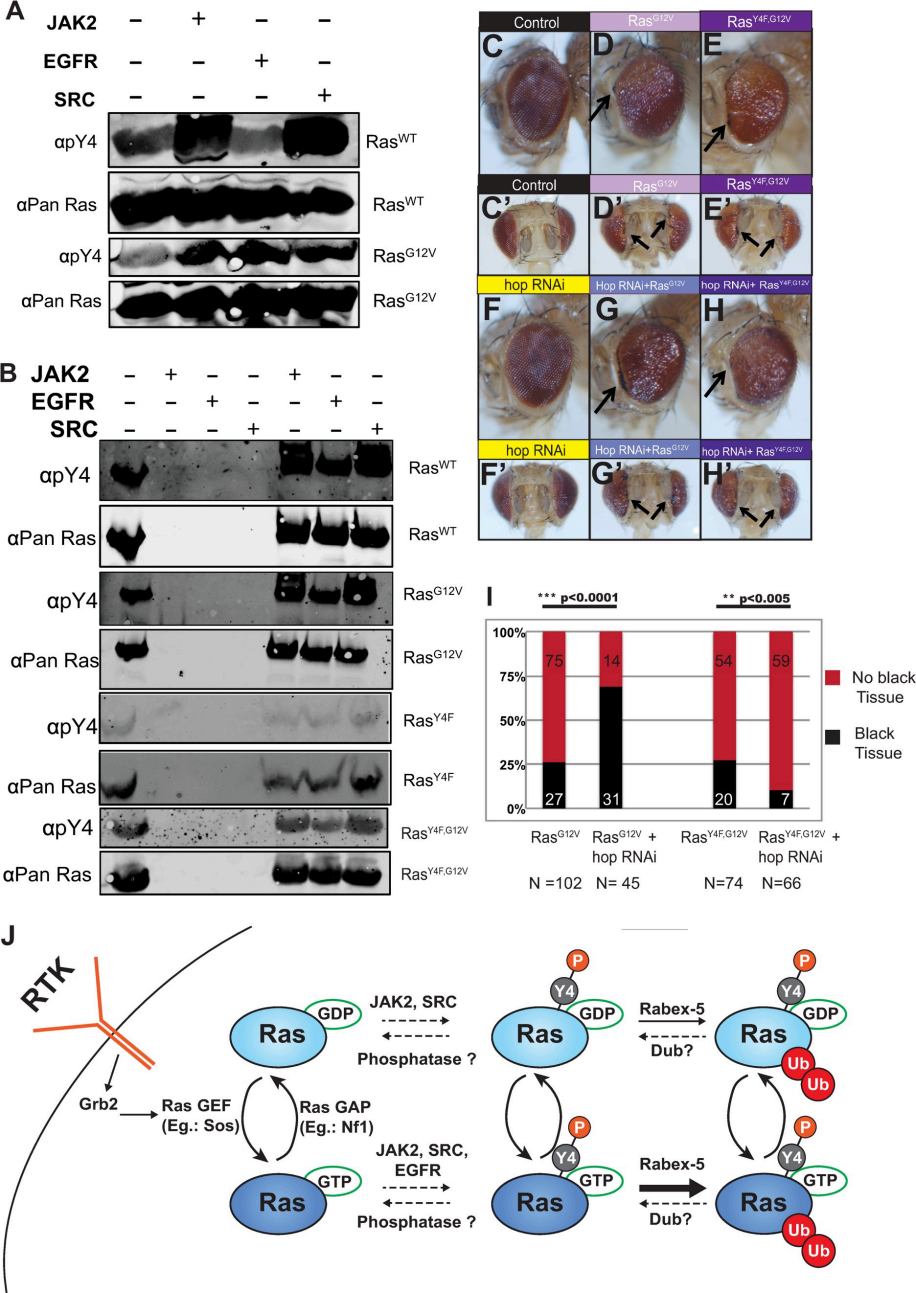
JAK2, SRC, and EGFR promote Ras phosphorylation at Y4. (A) Ras^WT^ and Ras^G12V^ proteins incubated in the presence or absence of JAK2, EGFR, or SRC proteins. Anti-pY4 antibodies recognize a baseline level of recombinant Ras protein species (lane 1). Increased recognition of Ras^WT^ protein by anti-pY4 antibodies is seen upon incubation with JAK2 (lane 2) and SRC (lane 4) but not EGFR (lane 3) compared to unmodified protein (lane 1). Increased recognition of Ras^G12V^ protein by anti-pY4 antibodies is seen upon incubation with JAK2 (lane 2), EGFR (lane 3) and SRC (lane 4) compared to unmodified protein (lane 1). (B) Ras^WT^, Ras^G12V^, Ras^Y4F^, and Ras^Y4F,G12V^ proteins incubated in the presence or absence of different preparations of JAK2, EGFR, or SRC proteins. Increased recognition of Ras^WT^ and Ras^G12V^ proteins by anti-pY4 antibodies is seen upon incubation with JAK2 (lane 5) and SRC (lane 7) but not EGFR (lane 6) compared to unmodified protein (lane 1). No difference in recognition of Ras^Y4F^ or Ras^Y4F,G12V^ proteins by anti-pY4 antibodies is seen upon incubation with JAK2 (lane 5), EGFR (lane 6) and SRC (lane 7) compared to unmodified protein (lane 1). Anti-pY4 antibodies also recognize JAK2 and SRC (S6B–S6H Fig) but this recognition does not interfere with detection of Ras proteins which run at a different size. (C-C’) Control *GMR-gal4/+* eye shown in profile (C) and from overhead (C’). (D-D’) Ras^G12V^ expressed using *GMR-gal4*. Eyes are rough and show some loss of eye pigment. Some eyes have black tissue at the periphery of the eye (arrow) shown in profile (D) and overhead (D’). (E-E-) Ras^Y4F,G12V^ expressed using *GMR-gal4*. Some eyes have black tissue at the periphery of the eye (arrow) shown in profile (E) and overhead (E’). (F-F’) *hop* RNAi driven by *GMR-gal4* yields no visible phenotype shown in profile (F) and overhead (F’). (G-G’) *hop* RNAi concurrent to Ras^G12V^ expression using *GMR-gal4*. Eyes are rough and show a more consistent appearance of black tissue (arrow, quantified in I) shown in profile (G) and overhead (G’). (H-H’) *hop* RNAi concurrent to Ras^Y4F,G12V^ expression using *GMR-gal4*. Eyes are rough but do not show enhancement of the black tissue (arrow, quantified in I) shown in profile (H) or overhead (H’). (I) Graph quantifying the presence of black tissue in control Ras^G12V^ and Ras^Y4F,G12V^ eyes or Ras^G12V^ and Ras^Y4F,G12V^ eyes undergoing concurrent *hop* RNAi. *hop* RNAi enhances the appearance of black tissue in Ras^G12V^ eyes but not in Ras^Y4F,G12V^ eyes. In the case shown, *hop* RNAi suppresses the appearance of black tissue in Ras^Y4F,G12V^ eyes. Suppression was reproducible but variable; in some trials we saw no statistically significant difference between Ras^Y4F,G12V^ eyes and Ras^Y4F,G12V^ eyes undergoing concurrent *hop* RNAi. Total N is indicated below the graph, and N for each category is indicated in each section of the bar graph.*** indicates p<0.0001, and ** indicates p<0.005 from CHITEST function in Excel for Chi-square statistical analysis comparing the percentage of black tissue between the indicated genotypes. Female eyes are shown in C-H and quantified in I. Increased lethality in males in these experiments resulted in numbers too small for statistical analysis. (J) We propose a model that Ras phosphorylation at Y4 promotes ubiquitination of Ras-GDP and Ras-GTP by Rabex-5. We consistently see greater ubiquitination of Ras^G12V^ than of Ras^WT^, and this is also seen for human Ras [12]. This finding, together with additional amino acids affecting ubiquitination of Ras^G12V^ than in Ras^WT^ (S2B Fig), suggest that there could be one kinase that targets both Ras-GDP and Ras-GTP and a second kinase that also targets Ras-GTP.

No motif or recognition signal responsible for targeting Ras for inhibition by Rabex-5 has previously been identified in Ras. Our findings demonstrate that Rabex-5-mediated inhibition of *Drosophila* Ras requires conserved Ras residue Tyrosine 4 *in vitro* and *in vivo* to regulate Ras. We cannot rule out a requirement for the tyrosine hydroxyl group in protein interactions contributing to this inhibition separate from its role in phosphorylation. However, to more specifically address phosphorylation of *Drosophila* Ras at Y4, we generated anti-phospho-Y4 (anti-pY4) rabbit polyclonal antibodies (described further in the methods). Anti-pY4 polyclonal antibodies recognized a number of proteins in cell lysates with presumably similar epitopes (S6A Fig), and they also recognized unphosphorylated Ras proteins expressed in and purified from bacteria at a baseline level (Fig 5A and 5B, S6B–S6H Fig). Baseline recognition of unmodified Ras proteins makes it difficult to assess subtle changes in phosphorylation of Y4 from cellular samples. Alanine scanning (S2 Fig) identified a role for Y4 and V7 in ubiquitination of both Ras^WT^ and Ras^G12V^; these residues represent a YXXV motif known to be recognized by tyrosine kinases JAK2 [32] and SRC [33–34]. Our alanine scanning also showed a role for E3 in Ras^G12V^ but not in Ras^WT^; EY is a motif recognized by EGFR [35]. Recombinant His6-tagged Ras^WT^ and Ras^G12V^ proteins purified from bacteria were incubated in the presence or absence of purchased recombinant active JAK2, SRC, and EGFR kinases. Reproducibly, anti-p-Y4 antibodies showed a dramatic increase in recognition of Ras^WT^ and Ras^G12V^ proteins compared to the baseline recognition of non-phosphorylated recombinant protein after incubation with JAK2 and SRC kinases (Fig 5A and 5B; S6B–S6F Fig). Anti-pY4 antibodies did not show a difference in recognition of Ras^WT^ protein versus Ras^WT^ protein incubated with EGFR kinase (Fig 5A and 5B, S6C Fig, S6E Fig) but showed dramatically increased recognition of Ras^G12V^ incubated with specific preparations of EGFR protein (Fig 5A, S6D Fig) but not with all EGFR preparations (Fig 5B, S6F Fig) compared to its recognition of Ras^G12V^ protein alone. To confirm that recognition depended on Y4, we repeated these assays with recombinant His6-tagged Ras^Y4F^ and Ras^Y4F,G12V^ proteins. Anti-pY4 antibodies did not show a difference in recognition between Ras^Y4F^ or Ras^Y4F,G12V^ proteins versus Ras^Y4F^ or Ras^Y4F,G12V^ proteins incubated with JAK2, SRC, or EGFR kinases (Fig 5B, S6G and S6H Fig). These findings are consistent with a model that JAK2 and SRC kinases are capable of promoting phosphorylation of Ras^WT^ and Ras^G12V^ at Y4 *in vitro*. Our results could also be consistent with a model that EGFR can promote phosphorylation of Ras^G12V^ at Y4 *in vitro*. However, given that this activity varied between preparations of commercial EGFR purified from HEK293 cells, it is possible that this activity relies on a co-purifying factor and is not intrinsic to EGFR. Alternatively, EGFR activity for Ras Y4 may require specific modifications of EGFR or co-factors not consistent between preparations. Anti-pY4 antibodies also recognized JAK2 and SRC kinases to varying degrees depending on specific preparations (S6C–S6H Fig), presumably due to the similar epitopes of their auto-phosphorylation sites with their site in the Ras N-terminus used to generate the anti-pY4 antibodies. Taken together, our *in vivo* phenotypic data (S3–S5 Figs) and *in vitro* kinase assay data (Fig 5, S6 Fig) are consistent with a model that the Y4 requirement involves phosphorylation potentially by JAK2, SRC, and/or EGFR tyrosine kinases (summarized schematically in Fig 5J). Serine and threonine phosphorylation have been reported to be required for recognition by the SCF family of cullin ring ligases [36; for review, 37–38]. Reports of tyrosine phosphorylation directing recognition by an E3 are relatively rare. A role for Y4 phosphorylation in inhibition by Rabex-5 could reflect an increased affinity of Rabex-5 for Ras phosphorylated at Y4 as with the SCF and its phosphorylated substrates. Alternatively, a phosphorylated tyrosine could serve another role such as recruitment of an SH2 domain containing adaptor protein that facilitates Ras interaction with Rabex-5.

### Reducing hop levels enhances Ras^G12V^ but not Ras^Y4F,G12V^

The Y4 kinase would be expected to increase inhibitory ubiquitination of Ras proteins, thus would serve to inhibit Ras biological outputs. JAK2, SRC, and EGFR have all been described to activate Ras signaling in various contexts. If one or more of these kinases also acts as the Y4 kinase to increase Rabex-5-mediated inhibitory Ras ubiquitination, this would serve as a feedback mechanism to ensure precise pathway outputs. Unfortunately, this also means that testing a role for these kinases to inhibit Ras *in vivo* using genetic interactions is challenging. Expressing EGFR (S7D Fig), Ras^G12V^ (Fig 5D and 5D’, S7B Fig), or Ras^Y4F,G12V^ (Fig 5E and 5E’, S7C Fig) individually in the eye using *GMR-gal4* leads to a rough eye, some loss of eye pigment, and in some eyes, the appearance of black tissue around the periphery of the eye compared to a control eye (Fig 5C and 5C’, S7A Fig). Indeed, co-expressing EGFR and Ras^G12V^ with *GMR-gal4* (S7E Fig) led to a dramatic enhancement of both the EGFR and the Ras^G12V^ rough eye phenotypes. Co-expression led to more significant loss of red eye pigment and an increased prevalence of black tissue at the periphery of the eye (S7E Fig). This differs from the phenotype of co-expressing EGFR and Ras^Y4F,G12V^ which also led to significant loss of eye pigment, but the black tissue phenotype appeared not only at the periphery of the eye but also in other regions of the eye (S7F Fig). The spread of black tissue from the periphery of the eye to other regions of the eye could reflect a greater enhancement of phenotype consistent with a role for EGFR to both restrict Ras^G12V^ activity and also to promote signaling through endogenous Ras but an inability to restrict Ras^Y4F,G12V^, or could reflect the enhanced phenotype of Ras^Y4F,G12V^ generally. Thus, these genetic interactions are difficult to interpret.

Low level RNAi of *hopscotch* (*hop*, an ortholog of JAK2) in the eye using *GMR-gal4* leads to no phenotype on its own (Fig 5F and 5F’) and resembles a control eye (Fig 5C and 5C’). RNAi to *hop* concurrent to expressing Ras^G12V^ (Fig 5G and 5G’, quantified in 5I, raw data in S3 File) consistently enhanced the phenotype of appearance of black tissue in the eye, a quantifiable phenotype we have used previously to reflect severity of phenotype [13] (Fig 5I). Importantly, this enhancement required Y4; we did not see enhancement of this black tissue phenotype upon *hop* RNAi concurrent to expressing Ras^Y4F,G12V^ (Fig 5H and 5H’, quantified in 5I). These *in vivo* interactions taken together with the *in vitro* kinase assays would be consistent with Hop/JAK2 activity normally acting to promote Y4 phosphorylation to restrict Ras activity in this context.

This report focuses on the importance of a specific tyrosine in directing Rabex-5 mediated ubiquitination of *Drosophila* Ras (model, Fig 5J). The site of inhibitory ubiquitination in *Drosophila* Ras and mammalian H-Ras and N-Ras has not been identified. There are a number of solvent-exposed lysines in mammalian Ras proteins reported to be sites of ubiquitination [20, 39–47], many of which are conserved in *Drosophila* Ras (summarized in S1 Table). K117 mono-ubiquitination has been reported to increase intrinsic nucleotide dissociation which facilitates GDP-GTP exchange, thus activating Ras. K147 mono-ubiquitination has been shown to decrease the rate of GTP hydrolysis mediated by GTPase Activating Proteins [41], which also activates Ras. K170 has been shown to serve as a site of LZTR1-mediated inhibitory ubiquitination [20]. We speculate that one of these other lysines could serve as the biologically relevant site of Rabex-5 mediated inhibitory mono- and di-ubiquitination of Ras proteins. In addition to the potential for inhibitory ubiquitination to promote re-localization of Ras (SS1I Fig and [15]), it is possible that Rabex-5 mediated inhibitory Ras ubiquitination decreases GDP-GTP exchange or increases the rate of GTP hydrolysis in opposition to the activating ubiquitination events at K117 and K147.

Importantly, Y4 mutations in H-Ras (HRas^Y4H^) have been reported in cerebellar glioblastomas [48]. H-Ras^Y4H^ tumor variants taken together with our work showing gain-of-function phenotypes upon mutation at Y4 suggest that Y4 phosphorylation is important for maintaining appropriate restriction of Ras activity and that mutation at Y4 evades such inhibition to promote tissue transformation.

## Materials and methods

### Reproducibility

The reported work represents reproducible experiments that reflect a minimum of three well-controlled, independent trials. For phenotypes that are subjective (not quantifiable), independent lab members scored progeny blind to avoid bias.

### Tissue culture

S2 cells were cultured using standard methods at 25°C in Schneider’s Drosophila Medium (1X) (Gibco, 21720–024). Cells were transfected using Effectene Transfection Reagent (Qiagen, Cat # 301427) according to manufacturer instructions with plasmids *pUAST-HA-Ub*, *Act-gal4*, *UAS myc Rabex-5*, *pIE*^*1-4*^ *Flag-His6-GFP Ras WT*, *pIE*^*1-4*^ *Flag-His6-GFP Ras 1-100CKML*, *pIE*^*1-4*^ *Flag-His6-GFP Ras 81–189*, *pIE*^*1-4*^ *Flag-His6-GFP Ras HVR*, *pIE*^*1-4*^ *Flag-His6-GFP Ras 180–189*, *pIE*^*1-4*^ *Flag-His6-GFP Ras 61-80CKML*, *pIE*^*1-4*^ *Flag-His6-GFP Ras 41-60CKML*, *pIE*^*1-4*^ *Flag-His6-GFP Ras 21-40CKML*, *pIE*^*1-4*^ *Flag-His6-GFP Ras 1-20CKML*, *pIE*^*1-4*^ *Flag-His6-GFP Ras 1–10 CKML*, *pIE*^*1-4*^ *Flag-His6-GFP Ras 1–60 CKML*, *pIE*^*1-4*^ *Flag-His6 Ras WT*, *pIE*^*1-4*^ *Flag-His6 Ras M1A*, *pIE*^*1-4*^ *Flag-His6 Ras T2A*, *pIE*^*1-4*^ *Flag-His6 Ras E3A*, *pIE*^*1-4*^ *Flag-His6 Ras Y4A*, *pIE*^*1-4*^ *Flag-His6 Ras Y4E*, *pIE*^*1-4*^ *Flag-His6 Ras Y4F*, *pIE*^*1-4*^ *Flag-His6 Ras Y4F*, *pIE*^*1-4*^ *Flag-His6 Ras E5A*, *pIE*^*1-4*^ *Flag-His6 Ras L6A*, *pIE*^*1-4*^ *Flag-His6 Ras V7A*, *pIE*^*1-4*^ *Flag-His6 Ras V8A*, *pIE*^*1-4*^ *Flag-His6 Ras V9A*, *pIE*^*1-4*^ *Flag-His6 Ras G10A*, *pIE*^*1-4*^ *Flag-His6 Ras M1A*,*G12V*, *pIE*^*1-4*^ *Flag-His6 Ras E3A*,*G12V*, *pIE*^*1-4*^ *Flag-His6 Ras Y4A*,*G12V*, *Flag-His6 Ras E5A*,*G12V*, *Flag-His6 Ras L6A*,*G12V*, *Flag-His6 Ras V7A*,*G12V*, *Flag-His6 Ras V8A*,*G12V*, *Flag-His6 Ras V9A*,*G12V*, *Flag-His6 Ras G10A*,*G12V* as indicated and harvested after 48–72 hours. Importantly, amounts of *pUAST-HA-Ub and Act-gal4* (to direct ubiquitin over-expression) were kept constant across experiments. Constructs are summarized in Table 1, and protein sequences of Ras constructs are detailed in Table 2.

**Table 1:** Table of reagents used in the manuscript with corresponding identifiers.

REAGENT or RESOURCE	SOURCE	IDENTIFIER
**Primary Antibodies**		
Mouse monoclonal anti-FLAG M2 primary antibodies	Sigma	Catalog #: F1804-5MG
Rabbit polyclonal anti-FLAG primary antibodies	Sigma	Catalog #: F7425-.2MG
Mouse monoclonal anti-HA primary antibodies	Roche	Catalog #: 12CA5
Rat monoclonal anti-Drosophila E Cadherin primary antibodies	Developmental Studies Hybridoma Bank	Catalog #: DCAD2
Mouse monoclonal anti-Pan Ras (AB-3) (Ras 10) primary antibodies	Millipore Sigma	Catalog #: OP40100UG
Mouse monoclonal anti-tubulin primary antibodies, Clone DM1A	Sigma	Catalog #: T9026.-.2ML
Mouse monoclonal anti-phospho-tyrosine primary antibodies, clone 4G10	Millipore Sigma	Catalog #: 05–321
Rabbit polyclonal anti- MTEphosphoYKLVV n(anti-Ras pY4)	Genscript	This study
**Secondary antibodies**		
Goat anti-mouse IgG (H+L) Alexa Fluor 488	Molecular Probes–Invitrogen	Catalog #: A11001
Goat anti-mouse IgG (H+L) Alexa Fluor 680	Molecular Probes–Invitrogen	Catalog #: A21057
Goat anti-rabbit IgG (H+L) Alexa Fluor 680	Molecular Probes–Invitrogen	Catalog #: A21076
Goat anti-mouse IgG (H+L) DyLight 800 Conjugated secondary antibodies	Thermo Scientific	Catalog #: 35521
Goat anti-rabbit IgG (H+L) DyLight 800 conjugated secondary antibodies	Thermo Scientific	Catalog #: 35571
**Cell Culture and biochemistry reagents**		
Qiagen Ni-NTA Agarose	QIAGEN	Catalog # 30230
COMPLETE EDTA free protease inhibitor Cocktail	Roche	Catalog # 11873580001
Phosphosafe Extraction Buffer	Sigma Aldrich	Catalog # 71296–3
Phenylmethylsulfonyl Fluoride	Sigma	Catalog # S-6508
Schneider’s Drosophila Medium (1X)	GIBCO	21720–024
Effectene Transfection Reagent	QIAGEN	Catalog # 301427
**Recombinant proteins**		
JAK2 JH1 active human Jak2 JH1 kinase	Sigma-Aldrich	Catalog # SPR0171-10UG
SRC, active human GST-tagged SRC	Sigma-Aldrich	Catalog #: S1076-10UG
EGFR/ERBB!, Human Egfr/ERBBB1	Sigma-Aldrich	Catalog #: SRP404-10UG
Rosetta-gami B(DE3)PLysS Competent cells	Novagen	Catalog# 71137–4
His6-Ras^WT^ (pet-28 Ras^WT^)	This study	
His6-Ras^Y4F^ (pet-28 Ras^Y4F^)	This study	
His6-Ras^G12V^ (pet-28 Ras^G12V^)	This study	
His6-Ras^Y4F,G12V^ (pet-28 Ras^Y4F,G12V^)	This study	
**Drosophila Strains**		
*w*^*1118*^	The fly community and Bloomington Drosophila Stock Center (BDSC)	BL-3605, BL-5905 and others RRID:BDSC_3605, RRID:BDSC_5905
*Tub-gal4*	The NYC fly community	Can be obtained from BDSC, BL-5138, RRID:BDSC_5138
*Act5C-gal4*	BDSC	BL3954, RRID:BDSC_3954
*ms1096-gal4*	BDSC	BL-8696 RRID:BDSC_8696
*c765-gal4*	BDSC, NYC fly community	Can be obtained from BDSC, BL-36523 RRID:BDSC_36523
*Ey-gal4*	NYC fly community	
*GMR-gal4*	BDSC	BL-8605, RRID:BDSC_8605
*He-gal4; UAS GFP*.*nls*	BDSC	BL-8700, RRID:BDSC_8700
*UAS hop*^*IR*^ (P{TRiP.JF01267})	BDSC	BL-31699; RRID:BDSC_31699
*UAS EGFR*	BDSC	BL-9535; RRID:BDSC_9535
*UAS Rabex-5*^*IR*^ (P{GD14133}v46329 CG9139GD14133)	VDRC	VDRCID dna14133
*UAS Rabex-5*^*DPYT*^	Yan et al. 2010 [13]	
*UAS Flag-His6-Ras*^*WT*^	This study	
*UAS Flag-His6-Ras*^*Y4E*^	This study	
*UAS Flag-His6-Ras*^*Y4F*^	This study	
*UAS Flag-His6-Ras*^*G12V*^	This study	
*UAS Flag-His6-Ras*^*Y4E*,*G12V*^	This study	
*UAS Flag-His6-Ras*^*Y4F*,*G12V*^	This study	
**Experimental Models: Cell Lines**		
*Drosophila* S2 cells	A gift from the NYC fly community	
**Recombinant DNA**		
pUAST-HA-Ub	Yan et al. 2010	
Act-gal4	A gift from the Mlodzik lab, also used in Yan et al. 2010	
UAS myc Rabex-5	Yan et al. 2010	
pIE^1-4^ Flag-His6-GFP Ras WT	This study	
pIE^1-4^ Flag-His6-GFP Ras 1-100CKML	This study	
pIE^1-4^ Flag-His6-GFP Ras 81–189	This study	
pIE^1-4^ Flag-His6-GFP Ras HVR	This study	
pIE^1-4^ Flag-His6-GFP Ras 180–189	This study	
pIE^1-4^ Flag-His6-GFP Ras 61-80CKML	This study	
pIE^1-4^ Flag-His6-GFP Ras 41-60CKML	This study	
pIE^1-4^ Flag-His6-GFP Ras 21-40CKML	This study	
pIE^1-4^ Flag-His6-GFP Ras 1-60CKML	This study	
pIE^1-4^ Flag-His6-GFP Ras 1-20CKML	This study	
pIE^1-4^ Flag-His6-GFP Ras 1–10 CKML	This study	
pIE^1-4^ MYC GFP 1–20 CKML	This study	
pIE^1-4^ MYC GFP 61–80 CKML	This study	
pIE^1-4^ Flag-His6-GFP Ras2	This study	
pIE^1-4^ Flag-His6 Ras WT	Yan et al. 2010	
pIE^1-4^ Flag-His6 Ras M1A	This study	
pIE^1-4^ Flag-His6 Ras T2A	This study	
pIE^1-4^ Flag-His6 Ras E3A	This study	
pIE^1-4^ Flag-His6 Ras Y4A	This study	
pIE^1-4^ Flag-His6 Ras Y4E	This study	
pIE^1-4^ Flag-His6 Ras Y4F	This study	
pIE^1-4^ Flag-His6 Ras E5A	This study	
pIE^1-4^ Flag-His6 Ras L6A	This study	
pIE^1-4^ Flag-His6 Ras V7A	This study	
pIE^1-4^ Flag-His6 Ras V8A	This study	
pIE^1-4^ Flag-His6 Ras V9A	This study	
pIE^1-4^ Flag-His6 Ras G10A	This study	
pIE^1-4^ Flag-His6 Ras G12V	This study	
pIE^1-4^ Flag-His6 Ras M1A,G12V	This study	
pIE^1-4^ Flag-His6 Ras T2A,G12V	This study	
pIE^1-4^ Flag-His6 Ras E3A,G12V	This study	
pIE^1-4^ Flag-His6 Ras Y4A,G12V	This study	
pIE^1-4^ Flag-His6 Ras K5A,G12V	This study	
pIE^1-4^ Flag-His6 Ras L6A,G12V	This study	
pIE^1-4^ Flag-His Ras V7A,G12V	This study	
pIE^1-4^ Flag-His6 Ras V8A,G12V	This study	
pIE^1-4^ Flag-His6 Ras V9A,G12V	This study	
pIE^1-4^ Flag-His6 Ras G10A,G12V	This study	
pet28-Ras^WT^	This study	
pet28-Ras^Y4F^	This study	
pet28-Ras^G12V^	This study	
pet28-Ras^Y4F,G12V^	This study	
**Software**		
Image J		https://imagej.nih.gov/ij/
Adobe Photoshop		https://www.adobe.com/products/photoshop.html
GraphPad Prism		https://www.graphpad.com/scientific-software/prism/
Microsoft Excel		https://www.microsoft.com/Microsoft/Excel/

**Table 2:** Table of protein sequences for Ras constructs used *in vitro* and *in vivo*.

Construct (as listed in Table 1)	Protein sequence (mutations bold, underlined; Tags and CAAX box, bolded): FLAG = DYKDDDDK, yellow His6 = HHHHHH GFP = MVSKGEELFTGVVPILVELDGDVNGHKFSVSGEGEGDATYGKLTLKFICTTGKLPVPWPTLVTTLTYGVQCFSRYPDHMKQHDFFKSAMPEGYVQERTIFFKDDGNYKTRAEVKFEGDTLVNRIELKGIDFKEDGNILGHKLEYNYNSHNVYIMADKQKNGIKVNFKIRHNIEDGSVQLADHYQQNTPIGDGPVLLPDNHYLSTQSALSKDPNEKRDHMVLLEFVTAAGITLGMDELYK MYC = EQKLISEEEDL Ras CAAX Box = CKML
pet28-Ras^WT^	MGSS**HHHHHH**SSGENLYFQGRPMTEYKLVVVGAGGVGKSALTIQLIQNHFVDEYDPTIEDSYRKQVVIDGETCLLDILDTAGQEEYSAMRDQYMRTGEGFLLVFAVNSAKSFEDIGTYREQIKRVKDAEEVPMVLVGNKCDLASWNVNNEQAREVAKQYGIPYIETSAKTRMGVDDAFYTLVREIRKDKDNKGRRGRKMNKPNRRFK**CKML**
pet28-Ras^Y4F^	MGSS**HHHHHH**SSGENLYFQGRPMTEFKLVVVGAGGVGKSALTIQLIQNHFVDEYDPTIEDSYRKQVVIDGETCLLDILDTAGQEEYSAMRDQYMRTGEGFLLVFAVNSAKSFEDIGTYREQIKRVKDAEEVPMVLVGNKCDLASWNVNNEQAREVAKQYGIPYIETSAKTRMGVDDAFYTLVREIRKDKDNKGRRGRKMNKPNRRFK**CKML**
pet28-Ras^G12V^	MGSS**HHHHHH**SSGENLYFQGRPMTEYKLVVVGAVGVGKSALTIQLIQNHFVDEYDPTIEDSYRKQVVIDGETCLLDILDTAGQEEYSAMRDQYMRTGEGFLLVFAVNSAKSFEDIGTYREQIKRVKDAEEVPMVLVGNKCDLASWNVNNEQAREVAKQYGIPYIETSAKTRMGVDDAFYTLVREIRKDKDNKGRRGRKMNKPNRRFK**CKML**
pet28-Ras^Y4F,G12V^	MGSS**HHHHHH**SSGENLYFQGRPMTEFKLVVVGAVGVGKSALTIQLIQNHFVDEYDPTIEDSYRKQVVIDGETCLLDILDTAGQEEYSAMRDQYMRTGEGFLLVFAVNSAKSFEDIGTYREQIKRVKDAEEVPMVLVGNKCDLASWNVNNEQAREVAKQYGIPYIETSAKTRMGVDDAFYTLVREIRKDKDNKGRRGRKMNKPNRRFK**CKML**
pIE^1-4^ Flag-His6-GFP Ras WT	M**DYKDDDDK**RGS**HHHHHH**A**MVSKGEELFTGVVPILVELDGDVNGHKFSVSGEGEGDATYGKLTLKFICTTGKLPVPWPTLVTTLTYGVQCFSRYPDHMKQHDFFKSAMPEGYVQERTIFFKDDGNYKTRAEVKFEGDTLVNRIELKGIDFKEDGNILGHKLEYNYNSHNVYIMADKQKNGIKVNFKIRHNIEDGSVQLADHYQQNTPIGDGPVLLPDNHYLSTQSALSKDPNEKRDHMVLLEFVTAAGITLGMDELYK**ALEMTEYKLVVVGAGGVGKSALTIQLIQNHFVDEYDPTIEDSYRKQVVIDGETCLLDILDTAGQEEYSAMRDQYMRTGEGFLLVFAVNSAKSFEDIGTYREQIKRVKDAEEVPMVLVGNKCDLASWNVNNEQAREVAKQYGIPYIETSAKTRMGVDDAFYTLVREIRKDKDNKGRRGRKMNKPNRRFK**CKML**
pIE^1-4^ Flag-His6-GFP Ras 1-100CKML	M**DYKDDDDK**RGS**HHHHHH**A**MVSKGEELFTGVVPILVELDGDVNGHKFSVSGEGEGDATYGKLTLKFICTTGKLPVPWPTLVTTLTYGVQCFSRYPDHMKQHDFFKSAMPEGYVQERTIFFKDDGNYKTRAEVKFEGDTLVNRIELKGIDFKEDGNILGHKLEYNYNSHNVYIMADKQKNGIKVNFKIRHNIEDGSVQLADHYQQNTPIGDGPVLLPDNHYLSTQSALSKDPNEKRDHMVLLEFVTAAGITLGMDELYK**ALEMTEYKLVVVGAGGVGKSALTIQLIQNHFVDEYDPTIEDSYRKQVVIDGETCLLDILDTAGQEEYSAMRDQYMRTGEGFLLVFAVNSAKSFEDIGTYREQI**CKML**
pIE^1-4^ Flag-His6-GFP Ras 81–189	M**DYKDDDDK**RGS**HHHHHH**A**MVSKGEELFTGVVPILVELDGDVNGHKFSVSGEGEGDATYGKLTLKFICTTGKLPVPWPTLVTTLTYGVQCFSRYPDHMKQHDFFKSAMPEGYVQERTIFFKDDGNYKTRAEVKFEGDTLVNRIELKGIDFKEDGNILGHKLEYNYNSHNVYIMADKQKNGIKVNFKIRHNIEDGSVQLADHYQQNTPIGDGPVLLPDNHYLSTQSALSKDPNEKRDHMVLLEFVTAAGITLGMDELYK**ALEVFAVNSAKSFEDIGTYREQIKRVKDAEEVPMVLVGNKCDLASWNVNNEQAREVAKQYGIPYIETSAKTRMGVDDAFYTLVREIRKDKDNKGRRGRKMNKPNRRFK**CKML**
pIE^1-4^ Flag-His6-GFP Ras HVR	M**DYKDDDDK**RGS**HHHHHH**A**MVSKGEELFTGVVPILVELDGDVNGHKFSVSGEGEGDATYGKLTLKFICTTGKLPVPWPTLVTTLTYGVQCFSRYPDHMKQHDFFKSAMPEGYVQERTIFFKDDGNYKTRAEVKFEGDTLVNRIELKGIDFKEDGNILGHKLEYNYNSHNVYIMADKQKNGIKVNFKIRHNIEDGSVQLADHYQQNTPIGDGPVLLPDNHYLSTQSALSKDPNEKRDHMVLLEFVTAAGITLGMDELYK**ALEDKDNKGRRGRKMNKPNRRFK**CKML**
pIE^1-4^ Flag-His6-GFP Ras 180–189	M**DYKDDDDK**RGS**HHHHHH**A**MVSKGEELFTGVVPILVELDGDVNGHKFSVSGEGEGDATYGKLTLKFICTTGKLPVPWPTLVTTLTYGVQCFSRYPDHMKQHDFFKSAMPEGYVQERTIFFKDDGNYKTRAEVKFEGDTLVNRIELKGIDFKEDGNILGHKLEYNYNSHNVYIMADKQKNGIKVNFKIRHNIEDGSVQLADHYQQNTPIGDGPVLLPDNHYLSTQSALSKDPNEKRDHMVLLEFVTAAGITLGMDELYK**ALEPNRRFK**CKML**
pIE^1-4^ Flag-His6-GFP Ras 61-80CKML	M**DYKDDDDK**RGS**HHHHHH**A**MVSKGEELFTGVVPILVELDGDVNGHKFSVSGEGEGDATYGKLTLKFICTTGKLPVPWPTLVTTLTYGVQCFSRYPDHMKQHDFFKSAMPEGYVQERTIFFKDDGNYKTRAEVKFEGDTLVNRIELKGIDFKEDGNILGHKLEYNYNSHNVYIMADKQKNGIKVNFKIRHNIEDGSVQLADHYQQNTPIGDGPVLLPDNHYLSTQSALSKDPNEKRDHMVLLEFVTAAGITLGMDELYK**ALEQEEYSAMRDQYMRTGEGFLL**CKML**
pIE^1-4^ Flag-His6-GFP Ras 41-60CKML	M**DYKDDDDK**RGS**HHHHHH**A**MVSKGEELFTGVVPILVELDGDVNGHKFSVSGEGEGDATYGKLTLKFICTTGKLPVPWPTLVTTLTYGVQCFSRYPDHMKQHDFFKSAMPEGYVQERTIFFKDDGNYKTRAEVKFEGDTLVNRIELKGIDFKEDGNILGHKLEYNYNSHNVYIMADKQKNGIKVNFKIRHNIEDGSVQLADHYQQNTPIGDGPVLLPDNHYLSTQSALSKDPNEKRDHMVLLEFVTAAGITLGMDELYK**ALERKQVVIDGETCLLDILDTAG**CKML**
pIE^1-4^ Flag-His6-GFP Ras 21-40CKML	M**DYKDDDDK**RGS**HHHHHH**A**MVSKGEELFTGVVPILVELDGDVNGHKFSVSGEGEGDATYGKLTLKFICTTGKLPVPWPTLVTTLTYGVQCFSRYPDHMKQHDFFKSAMPEGYVQERTIFFKDDGNYKTRAEVKFEGDTLVNRIELKGIDFKEDGNILGHKLEYNYNSHNVYIMADKQKNGIKVNFKIRHNIEDGSVQLADHYQQNTPIGDGPVLLPDNHYLSTQSALSKDPNEKRDHMVLLEFVTAAGITLGMDELYK**ALEIQLIQNHFVDEYDPTIEDSY**CKML**
pIE^1-4^ Flag-His6-GFP Ras 1-60CKML	M**DYKDDDDK**RGS**HHHHHH**A**MVSKGEELFTGVVPILVELDGDVNGHKFSVSGEGEGDATYGKLTLKFICTTGKLPVPWPTLVTTLTYGVQCFSRYPDHMKQHDFFKSAMPEGYVQERTIFFKDDGNYKTRAEVKFEGDTLVNRIELKGIDFKEDGNILGHKLEYNYNSHNVYIMADKQKNGIKVNFKIRHNIEDGSVQLADHYQQNTPIGDGPVLLPDNHYLSTQSALSKDPNEKRDHMVLLEFVTAAGITLGMDELYK**ALEMTEYKLVVVGAGGVGKSALTIQLIQNHFVDEYDPTIEDSYRKQVVIDGETCLLDILDTAG**CKML**
pIE^1-4^ Flag-His6-GFP Ras 1-20CKML	M**DYKDDDDK**RGS**HHHHHH**A**MVSKGEELFTGVVPILVELDGDVNGHKFSVSGEGEGDATYGKLTLKFICTTGKLPVPWPTLVTTLTYGVQCFSRYPDHMKQHDFFKSAMPEGYVQERTIFFKDDGNYKTRAEVKFEGDTLVNRIELKGIDFKEDGNILGHKLEYNYNSHNVYIMADKQKNGIKVNFKIRHNIEDGSVQLADHYQQNTPIGDGPVLLPDNHYLSTQSALSKDPNEKRDHMVLLEFVTAAGITLGMDELYK**ALEMTEYKLVVVGAGGVGKSALT**CKML**
pIE^1-4^ Flag-His6-GFP Ras 1–10 CKML	M**DYKDDDDK**RGS**HHHHHH**A**MVSKGEELFTGVVPILVELDGDVNGHKFSVSGEGEGDATYGKLTLKFICTTGKLPVPWPTLVTTLTYGVQCFSRYPDHMKQHDFFKSAMPEGYVQERTIFFKDDGNYKTRAEVKFEGDTLVNRIELKGIDFKEDGNILGHKLEYNYNSHNVYIMADKQKNGIKVNFKIRHNIEDGSVQLADHYQQNTPIGDGPVLLPDNHYLSTQSALSKDPNEKRDHMVLLEFVTAAGITLGMDELYK**ALEMTEYKLVVVG**CKML**
pIE^1-4^ MYC GFP 1–20 CKML	M**EQKLISEEDL**A**MVSKGEELFTGVVPILVELDGDVNGHKFSVSGEGEGDATYGKLTLKFICTTGKLPVPWPTLVTTLTYGVQCFSRYPDHMKQHDFFKSAMPEGYVQERTIFFKDDGNYKTRAEVKFEGDTLVNRIELKGIDFKEDGNILGHKLEYNYNSHNVYIMADKQKNGIKVNFKIRHNIEDGSVQLADHYQQNTPIGDGPVLLPDNHYLSTQSALSKDPNEKRDHMVLLEFVTAAGITLGMDELYK**GGLEMTEYKLVVVGAGGVGKSALT**CKML**
pIE^1-4^ MYC GFP 61–80 CKML	ME**QKLISEEDL**A**MVSKGEELFTGVVPILVELDGDVNGHKFSVSGEGEGDATYGKLTLKFICTTGKLPVPWPTLVTTLTYGVQCFSRYPDHMKQHDFFKSAMPEGYVQERTIFFKDDGNYKTRAEVKFEGDTLVNRIELKGIDFKEDGNILGHKLEYNYNSHNVYIMADKQKNGIKVNFKIRHNIEDGSVQLADHYQQNTPIGDGPVLLPDNHYLSTQSALSKDPNEKRDHMVLLEFVTAAGITLGMDELYK**GGLEQEEYSAMRDQYMRTGEGFLL**CKML**
pIE^1-4^ Flag-His6 Ras WT	M**DYKDDDDK**RGS**HHHHHH**ALEMTEYKLVVVGAGGVGKSALTIQLIQNHFVDEYDPTIEDSYRKQVVIDGETCLLDILDTAGQEEYSAMRDQYMRTGEGFLLVFAVNSAKSFEDIGTYREQIKRVKDAEEVPMVLVGNKCDLASWNVNNEQAREVAKQYGIPYIETSAKTRMGVDDAFYTLVREIRKDKDNKGRRGRKMNKPNRRFK**CKML**
pIE^1-4^ Flag-His6 Ras M1A	M**DYKDDDDK**RGS**HHHHHH****A**LEATEYKLVVVGAGGVGKSALTIQLIQNHFVDEYDPTIEDSYRKQVVIDGETCLLDILDTAGQEEYSAMRDQYMRTGEGFLLVFAVNSAKSFEDIGTYREQIKRVKDAEEVPMVLVGNKCDLASWNVNNEQAREVAKQYGIPYIETSAKTRMGVDDAFYTLVREIRKDKDNKGRRGRKMNKPNRRFK**CKML**
pIE^1-4^ Flag-His6 Ras T2A	M**DYKDDDDK**RGS**HHHHHH**ALEM**A**EYKLVVVGAGGVGKSALTIQLIQNHFVDEYDPTIEDSYRKQVVIDGETCLLDILDTAGQEEYSAMRDQYMRTGEGFLLVFAVNSAKSFEDIGTYREQIKRVKDAEEVPMVLVGNKCDLASWNVNNEQAREVAKQYGIPYIETSAKTRMGVDDAFYTLVREIRKDKDNKGRRGRKMNKPNRRFK**CKML**
pIE^1-4^ Flag-His6 Ras E3A	M**DYKDDDDK**RGS**HHHHHH**ALEMT**A**YKLVVVGAGGVGKSALTIQLIQNHFVDEYDPTIEDSYRKQVVIDGETCLLDILDTAGQEEYSAMRDQYMRTGEGFLLVFAVNSAKSFEDIGTYREQIKRVKDAEEVPMVLVGNKCDLASWNVNNEQAREVAKQYGIPYIETSAKTRMGVDDAFYTLVREIRKDKDNKGRRGRKMNKPNRRFK**CKML**
pIE^1-4^ Flag-His6 Ras Y4A	M**DYKDDDDK**RGS**HHHHHH**ALEMTE**A**KLVVVGAGGVGKSALTIQLIQNHFVDEYDPTIEDSYRKQVVIDGETCLLDILDTAGQEEYSAMRDQYMRTGEGFLLVFAVNSAKSFEDIGTYREQIKRVKDAEEVPMVLVGNKCDLASWNVNNEQAREVAKQYGIPYIETSAKTRMGVDDAFYTLVREIRKDKDNKGRRGRKMNKPNRRFK**CKML**
pIE^1-4^ Flag-His6 Ras Y4E	M**DYKDDDDK**RGS**HHHHHH**ALEMTE**E**KLVVVGAGGVGKSALTIQLIQNHFVDEYDPTIEDSYRKQVVIDGETCLLDILDTAGQEEYSAMRDQYMRTGEGFLLVFAVNSAKSFEDIGTYREQIKRVKDAEEVPMVLVGNKCDLASWNVNNEQAREVAKQYGIPYIETSAKTRMGVDDAFYTLVREIRKDKDNKGRRGRKMNKPNRRFK**CKML**
pIE^1-4^ Flag-His6 Ras Y4F	M**DYKDDDDK**RGS**HHHHHH**ALEMTE**F**KLVVVGAGGVGKSALTIQLIQNHFVDEYDPTIEDSYRKQVVIDGETCLLDILDTAGQEEYSAMRDQYMRTGEGFLLVFAVNSAKSFEDIGTYREQIKRVKDAEEVPMVLVGNKCDLASWNVNNEQAREVAKQYGIPYIETSAKTRMGVDDAFYTLVREIRKDKDNKGRRGRKMNKPNRRFK**CKML**
pIE^1-4^ Flag-His6 Ras E5A	M**DYKDDDDK**RGS**HHHHHH**ALEMTEY**A**LVVVGAGGVGKSALTIQLIQNHFVDEYDPTIEDSYRKQVVIDGETCLLDILDTAGQEEYSAMRDQYMRTGEGFLLVFAVNSAKSFEDIGTYREQIKRVKDAEEVPMVLVGNKCDLASWNVNNEQAREVAKQYGIPYIETSAKTRMGVDDAFYTLVREIRKDKDNKGRRGRKMNKPNRRFK**CKML**
pIE^1-4^ Flag-His6 Ras L6A	M**DYKDDDDK**RGS**HHHHHH**ALEMTEYK**A**VVVGAGGVGKSALTIQLIQNHFVDEYDPTIEDSYRKQVVIDGETCLLDILDTAGQEEYSAMRDQYMRTGEGFLLVFAVNSAKSFEDIGTYREQIKRVKDAEEVPMVLVGNKCDLASWNVNNEQAREVAKQYGIPYIETSAKTRMGVDDAFYTLVREIRKDKDNKGRRGRKMNKPNRRFK**CKML**
pIE^1-4^ Flag-His6 Ras V7A	M**DYKDDDDK**RGS**HHHHHH**ALEMTEYKL**A**VVGAGGVGKSALTIQLIQNHFVDEYDPTIEDSYRKQVVIDGETCLLDILDTAGQEEYSAMRDQYMRTGEGFLLVFAVNSAKSFEDIGTYREQIKRVKDAEEVPMVLVGNKCDLASWNVNNEQAREVAKQYGIPYIETSAKTRMGVDDAFYTLVREIRKDKDNKGRRGRKMNKPNRRFK**CKML**
pIE^1-4^ Flag-His6 Ras V8A	M**DYKDDDDK**RGS**HHHHHH**ALEMTEYKLV**A**VGAGGVGKSALTIQLIQNHFVDEYDPTIEDSYRKQVVIDGETCLLDILDTAGQEEYSAMRDQYMRTGEGFLLVFAVNSAKSFEDIGTYREQIKRVKDAEEVPMVLVGNKCDLASWNVNNEQAREVAKQYGIPYIETSAKTRMGVDDAFYTLVREIRKDKDNKGRRGRKMNKPNRRFK**CKML**
pIE^1-4^ Flag-His6 Ras V9A	M**DYKDDDDK**RGS**HHHHHH**ALEMTEYKLVV**A**GAGGVGKSALTIQLIQNHFVDEYDPTIEDSYRKQVVIDGETCLLDILDTAGQEEYSAMRDQYMRTGEGFLLVFAVNSAKSFEDIGTYREQIKRVKDAEEVPMVLVGNKCDLASWNVNNEQAREVAKQYGIPYIETSAKTRMGVDDAFYTLVREIRKDKDNKGRRGRKMNKPNRRFK**CKML**
pIE^1-4^ Flag-His6 Ras G10A	M**DYKDDDDK**RGS**HHHHHH**ALEMTEYKLVVV**A**AGGVGKSALTIQLIQNHFVDEYDPTIEDSYRKQVVIDGETCLLDILDTAGQEEYSAMRDQYMRTGEGFLLVFAVNSAKSFEDIGTYREQIKRVKDAEEVPMVLVGNKCDLASWNVNNEQAREVAKQYGIPYIETSAKTRMGVDDAFYTLVREIRKDKDNKGRRGRKMNKPNRRFK**CKML**
pIE^1-4^ Flag-His6 Ras G12V	M**DYKDDDDK**RGS**HHHHHH**ALEMTEYKLVVVGA**V**GVGKSALTIQLIQNHFVDEYDPTIEDSYRKQVVIDGETCLLDILDTAGQEEYSAMRDQYMRTGEGFLLVFAVNSAKSFEDIGTYREQIKRVKDAEEVPMVLVGNKCDLASWNVNNEQAREVAKQYGIPYIETSAKTRMGVDDAFYTLVREIRKDKDNKGRRGRKMNKPNRRFK**CKML**
pIE^1-4^ Flag-His6 Ras M1A,G12V	M**DYKDDDDK**RGS**HHHHHH****A**LEATEYKLVVVGA**V**GVGKSALTIQLIQNHFVDEYDPTIEDSYRKQVVIDGETCLLDILDTAGQEEYSAMRDQYMRTGEGFLLVFAVNSAKSFEDIGTYREQIKRVKDAEEVPMVLVGNKCDLASWNVNNEQAREVAKQYGIPYIETSAKTRMGVDDAFYTLVREIRKDKDNKGRRGRKMNKPNRRFK**CKML**
pIE^1-4^ Flag-His6 Ras T2A,G12V	M**DYKDDDDK**RGS**HHHHHH**ALEM**A**EYKLVVVGA**V**GVGKSALTIQLIQNHFVDEYDPTIEDSYRKQVVIDGETCLLDILDTAGQEEYSAMRDQYMRTGEGFLLVFAVNSAKSFEDIGTYREQIKRVKDAEEVPMVLVGNKCDLASWNVNNEQAREVAKQYGIPYIETSAKTRMGVDDAFYTLVREIRKDKDNKGRRGRKMNKPNRRFK**CKML**
pIE^1-4^ Flag-His6 Ras E3A,G12V	M**DYKDDDDK**RGS**HHHHHH**ALEMT**A**YKLVVVGA**V**GVGKSALTIQLIQNHFVDEYDPTIEDSYRKQVVIDGETCLLDILDTAGQEEYSAMRDQYMRTGEGFLLVFAVNSAKSFEDIGTYREQIKRVKDAEEVPMVLVGNKCDLASWNVNNEQAREVAKQYGIPYIETSAKTRMGVDDAFYTLVREIRKDKDNKGRRGRKMNKPNRRFK**CKML**
pIE^1-4^ Flag-His6 Ras Y4A,G12V	M**DYKDDDDK**RGS**HHHHHH**ALEMTE**A**KLVVVGA**V**GVGKSALTIQLIQNHFVDEYDPTIEDSYRKQVVIDGETCLLDILDTAGQEEYSAMRDQYMRTGEGFLLVFAVNSAKSFEDIGTYREQIKRVKDAEEVPMVLVGNKCDLASWNVNNEQAREVAKQYGIPYIETSAKTRMGVDDAFYTLVREIRKDKDNKGRRGRKMNKPNRRFK**CKML**
pIE^1-4^ Flag-His6 Ras K5A,G12V	M**DYKDDDDK**RGS**HHHHHH**ALEMTEY**A**LVVVGA**V**GVGKSALTIQLIQNHFVDEYDPTIEDSYRKQVVIDGETCLLDILDTAGQEEYSAMRDQYMRTGEGFLLVFAVNSAKSFEDIGTYREQIKRVKDAEEVPMVLVGNKCDLASWNVNNEQAREVAKQYGIPYIETSAKTRMGVDDAFYTLVREIRKDKDNKGRRGRKMNKPNRRFK**CKML**
pIE^1-4^ Flag-His6 Ras L6A,G12V	M**DYKDDDDK**RGS**HHHHHH**ALEMTEYK**A**VVVGA**V**GVGKSALTIQLIQNHFVDEYDPTIEDSYRKQVVIDGETCLLDILDTAGQEEYSAMRDQYMRTGEGFLLVFAVNSAKSFEDIGTYREQIKRVKDAEEVPMVLVGNKCDLASWNVNNEQAREVAKQYGIPYIETSAKTRMGVDDAFYTLVREIRKDKDNKGRRGRKMNKPNRRFK**CKML**
pIE^1-4^ Flag-His Ras V7A,G12V	M**DYKDDDDK**RGS**HHHHHH**ALEMTEYKL**A**VVGA**V**GVGKSALTIQLIQNHFVDEYDPTIEDSYRKQVVIDGETCLLDILDTAGQEEYSAMRDQYMRTGEGFLLVFAVNSAKSFEDIGTYREQIKRVKDAEEVPMVLVGNKCDLASWNVNNEQAREVAKQYGIPYIETSAKTRMGVDDAFYTLVREIRKDKDNKGRRGRKMNKPNRRFK**CKML**
pIE^1-4^ Flag-His6 Ras V8A,G12V	M**DYKDDDDK**RGS**HHHHHH**ALEMTEYKLV**A**VGA**V**GVGKSALTIQLIQNHFVDEYDPTIEDSYRKQVVIDGETCLLDILDTAGQEEYSAMRDQYMRTGEGFLLVFAVNSAKSFEDIGTYREQIKRVKDAEEVPMVLVGNKCDLASWNVNNEQAREVAKQYGIPYIETSAKTRMGVDDAFYTLVREIRKDKDNKGRRGRKMNKPNRRFK**CKML**
pIE^1-4^ Flag-His6 Ras V9A,G12V	M**DYKDDDDK**RGS**HHHHHH**ALEMTEYKLVV**A**GA**V**GVGKSALTIQLIQNHFVDEYDPTIEDSYRKQVVIDGETCLLDILDTAGQEEYSAMRDQYMRTGEGFLLVFAVNSAKSFEDIGTYREQIKRVKDAEEVPMVLVGNKCDLASWNVNNEQAREVAKQYGIPYIETSAKTRMGVDDAFYTLVREIRKDKDNKGRRGRKMNKPNRRFK**CKML**
pIE^1-4^ Flag-His6 Ras G10A,G12V	M**DYKDDDDK**RGS**HHHHHH**ALEMTEYKLVVV**A**A**V**GVGKSALTIQLIQNHFVDEYDPTIEDSYRKQVVIDGETCLLDILDTAGQEEYSAMRDQYMRTGEGFLLVFAVNSAKSFEDIGTYREQIKRVKDAEEVPMVLVGNKCDLASWNVNNEQAREVAKQYGIPYIETSAKTRMGVDDAFYTLVREIRKDKDNKGRRGRKMNKPNRRFK**CKML**
UAS Flag-His6-Ras^WT^	M**DYKDDDDK**RGS**HHHHHH**ALEMTEYKLVVVGAGGVGKSALTIQLIQNHFVDEYDPTIEDSYRKQVVIDGETCLLDILDTAGQEEYSAMRDQYMRTGEGFLLVFAVNSAKSFEDIGTYREQIKRVKDAEEVPMVLVGNKCDLASWNVNNEQAREVAKQYGIPYIETSAKTRMGVDDAFYTLVREIRKDKDNKGRRGRKMNKPNRRFK**CKML**
UAS Flag-His6-Ras^Y4E^	M**DYKDDDDK**RGS**HHHHHH**ALEMTE**E**KLVVVGAGGVGKSALTIQLIQNHFVDEYDPTIEDSYRKQVVIDGETCLLDILDTAGQEEYSAMRDQYMRTGEGFLLVFAVNSAKSFEDIGTYREQIKRVKDAEEVPMVLVGNKCDLASWNVNNEQAREVAKQYGIPYIETSAKTRMGVDDAFYTLVREIRKDKDNKGRRGRKMNKPNRRFK**CKML**
UAS Flag-His6-Ras^Y4F^	**M**DYKDDDDK**RGS**HHHHHH**ALEMTE****F****KLVVVGAGGVGKSALTIQLIQNHFVDEYDPTIEDSYRKQVVIDGETCLLDILDTAGQEEYSAMRDQYMRTGEGFLLVFAVNSAKSFEDIGTYREQIKRVKDAEEVPMVLVGNKCDLASWNVNNEQAREVAKQYGIPYIETSAKTRMGVDDAFYTLVREIRKDKDNKGRRGRKMNKPNRRFKCKML**
UAS Flag-His6-Ras^G12V^	M**DYKDDDDK**RGS**HHHHHH**ALEMTEYKLVVVGA**V**GVGKSALTIQLIQNHFVDEYDPTIEDSYRKQVVIDGETCLLDILDTAGQEEYSAMRDQYMRTGEGFLLVFAVNSAKSFEDIGTYREQIKRVKDAEEVPMVLVGNKCDLASWNVNNEQAREVAKQYGIPYIETSAKTRMGVDDAFYTLVREIRKDKDNKGRRGRKMNKPNRRFK**CKML**
UAS Flag-His6-Ras^Y4E,G12V^	M**DYKDDDDK**RGS**HHHHHH**ALEMTE**E**KLVVVGA**V**GVGKSALTIQLIQNHFVDEYDPTIEDSYRKQVVIDGETCLLDILDTAGQEEYSAMRDQYMRTGEGFLLVFAVNSAKSFEDIGTYREQIKRVKDAEEVPMVLVGNKCDLASWNVNNEQAREVAKQYGIPYIETSAKTRMGVDDAFYTLVREIRKDKDNKGRRGRKMNKPNRRFK**CKML**
UAS Flag-His6-Ras^Y4F,G12V^	M**DYKDDDDK**RGS**HHHHHH**ALEMTE**F**KLVVVGA**V**GVGKSALTIQLIQNHFVDEYDPTIEDSYRKQVVIDGETCLLDILDTAGQEEYSAMRDQYMRTGEGFLLVFAVNSAKSFEDIGTYREQIKRVKDAEEVPMVLVGNKCDLASWNVNNEQAREVAKQYGIPYIETSAKTRMGVDDAFYTLVREIRKDKDNKGRRGRKMNKPNRRFK**CKML**

### Schneider S2 cell extract preparation

Schneider S2 cell extracts were prepared immediately from harvested cells or from frozen cell pellets. Lysates were prepared in lysis buffer, 1X NP40 buffer (50 mM HEPES pH 7.4, 1% NP40, 1mM EDTA, 150 mM NaCl), 8M Urea, 5 mM beta-mercaptoethanol, 10 mM imidazole supplemented with Roche protease inhibitor cocktail and 1mM each of PMSF and Sodium Orthovanadate) and used in pull down assays described above or analyzed by Western.

### Nickel pull down of tagged Ras from Schneider S2 cells (for Western analysis)

Ni-NTA agarose beads (Qiagen) were equilibrated as per manufacturer’s instructions and then washed twice with binding buffer, pH 8.0 (1X PBS, 8 M Urea, 0.5% NP40, 10 mM Imidazole, 360 mM NaCl) followed by incubation with binding buffer, pH 8.0 supplemented with 1g/ml BSA. Lysates were then incubated with beads on a nutator for 2h at 4°C. Beads were then transferred to mini-columns on a vacuum assembly and washed 3X with wash buffer pH 6.3 (1X PBS, 8 M Urea, 0.5% NP40, 30 mM Imidazole, 360 mM NaCl). Purified His-tagged proteins were eluted from the beads by washing with elution buffer pH 6.3 (1X PBS, 8 M Urea, 0.5% NP40, 500 mM Imidazole, 360 mM NaCl). Eluate was boiled in 1X loading buffer for 10 minutes before storage at -20°C.

### Larval extract preparation

Extracts were prepared from larvae of the specified genotypes in Phosphosafe Extraction Reagent (Novagen) supplemented with protease inhibitors (Complete Phosphatase Inhibitor Cocktail, 1mM PMSF and 1mM Sodium Orthovanadate). Lysates of individual larvae were loaded per lane to demonstrate transgene protein levels.

### Development of anti-phospho-Y4 antibodies

A project was initiated with Genscript to produce MTE{pY}KLVVVGC peptides to immunize rabbits. Genscript immunized rabbits and purified phospho-specific polyclonal antisera which recognized MTE{pY}KLVVVGC peptides and delivered purified antibodies to us following purification. Antibodies were tested against cell lysates, larval lysates, and purified recombinant proteins.

### Western Blot analysis

Western blots used Immobilon-FL Transfer Membrane (Millipore, Cat # IPFL00010) and were visualized using the Li-Cor Odyssey System. Primary antibodies were anti-pY4 (rabbit polyclonal, this study; 1:2000); anti-FLAG (rabbit, Sigma Catalog # F7425-.2MG, 1:1000), anti-Pan Ras (mouse, Millipore Sigma Catalog # OP40100UG, 1:1000), anti-FLAG M2 (mouse, Sigma Catalog # F1804-5MG, 1:1000), anti-HA (mouse, Roche Catalog # 12CA5, 1:1000), anti-alpha tubulin (mouse, Sigma T9026.-.2ML, 1:8000), anti-phospho-tyrosine 4G10 (mouse, EMD Millipore Catalog # 05–321, 1:1000); secondary antibodies were Alexa Fluor goat anti-mouse 488 (Invitrogen, Catalog # A11001, 1:10,000), Alexa Fluor goat anti-rabbit 680 (Invitrogen, Catalog # A21076, 1:10,000), Alexa Fluor goat anti-mouse 680 (Invitrogen, Catalog # A21057, 1:10,000), DyLight goat anti-mouse 800 (Thermo Scientific, Catalog # 35521, 1:10,000), and DyLight goat anti-rabbit 800 (Thermo Scientific, Catalog # 35571, 1:10,000).

### Recombinant proteins

Ras^WT^ and Ras mutants (Ras^Y4F^, Ras^G12V^, and Ras^Y4F,G12V^) were cloned into pet-28 vectors for bacterial expression and purification of His6-tagged proteins. Proteins were expressed from these plasmids in Rosetta-gami B(DE3)PLysS strains (Novagen, 711374) following induction by IPTG. Proteins were purified on nickel beads. Protein sequences of Ras mutants are detailed in Table 2.

### Kinase assays

Active JAK2 (SRP0171), EGFR (SRP6404), and SRC (S1076) recombinant proteins were purchased from Sigma. Recombinant Ras^WT^ or Ras mutants on beads were incubated in the presence or absence of 100 micrograms (S6B Fig) or 20 micrograms (Fig 5A and 5B, S6C–S6H Fig) recombinant kinase proteins in kinase assay buffer (25mM TrisHCl pH7.5, 10mM MgCl_2_) for thirty minutes, quenched with sample buffer, run on a gel, and analyzed by Western.

### Western quantification and adjustment

Raw tiff files from the Li-Cor Odyssey were split from full color into specific channels corresponding to single antibodies that were then converted to grayscale using Adobe Photoshop. Images were cropped to fit figure panels; adjustments to brightness and contrast were applied uniformly to the entire images not to portions of an image. Quantification of percent ubiquitination utilized gels in which signals were not over-saturated. To quantify percent ubiquitination, gel lanes were analyzed using Image J; we summed the signal of unconjugated and ubiquitinated Ras bands in a single lane to define total Ras signal in that lane and then calculated the proportion of each band per total to indicate percent conjugated to ubiquitin out of total Ras. In this manner, we could make comparisons of percent conjugation to ubiquitin in one lane to the percent conjugation to ubiquitin in another lane from different lanes on the same gel within the same experiment.

### *Drosophila* experiments

Ras constructs were cloned into pUAST-attB with the FLAG and His6 sequences of MDYKDDDDKRGSHHHHHHALE preceding the Ras coding sequence. UAS Flag-His6-Ras^WT^, UAS Flag-His6-Ras^Y4E^, UAS Flag-His6-Ras^Y4F^, UAS Flag-His6-Ras^G12V^, UAS Flag-His6-Ras^Y4E,G12V^, and UAS Flag-His6-Ras^Y4F,G12V^ plasmids were sent to BestGene for injection and generation of transgenic lines at the attp40 locus. Lines were balanced over CyO or SM6-TM6B balancers and then maintained as true-breeding homozygous stocks. Genomic DNA was sequenced to confirm each insert. Gal4 drivers were obtained from the Bloomington Drosophila Stock center or other labs in the Drosophila community. *UAS hop*^*IR*^ (P{TRiP.JF01267}), *UAS EGFR* were from the Bloomington Stock center. UAS Rabex-5^IR^ (P{GD14133}v46329 CG9139GD14133) was obtained from the VDRC, VDRCID dna14133 and was characterized in our previous study [13]. Crosses were performed at the indicated temperatures on standard *Drosophila* medium. Raw wing images were converted to grayscale using Adobe Photoshop. Brightness and contrast of eye and wing images were adjusted using Adobe Photoshop to maximize clarity; adjustments were applied to the entire images. Genotypes are summarized below, and identifiers are annotated in Table 1. Protein sequences for Ras transgenes are detailed in Table 2.

Genotypes of fly images (Figures and Supplemental Figures):

*w; Tub-gal4/+* (Fig 2A and 2A’; S3C Fig, S3C’ Fig)

*w; UAS Ras*^*WT*^*/+; Tub-gal4/+* (Fig 2B and 2B’; S3D Fig, S3D’ Fig)

*w; UAS Ras*^*Y4F*^*/+; Tub-gal4/+* (Fig 2C and 2C’; S3E Fig, S3E’ Fig)

*MS1096-gal4* (Fig 2D; S2F Fig; Fig 4P, S5Q Fig)

*MS1096-gal4; UAS Ras*^*WT*^ (Fig 2E; S3G Fig)

*MS1096-gal4; UAS Ras*^*Y4F*^ (Fig 2F; S3H Fig)

*w; c765-gal4/+* (Fig 3A, 3D and 3G, S3A Fig, S3D Fig, S3G Fig; Fig 4M and 4R, S5N Fig, S5S Fig)

*w; UAS Ras*^*G12V*^*/+; c765-gal4/+* (Fig 3B, 3E and 3H, S4B Fig, S4E Fig, S4H Fig; Fig 4N, S5O Fig)

*w; UAS Rabex-5*^*DPYT*^*/+; c765gal4/+* (Fig 3D’, S4D’ Fig)

*w; UAS Ras*^*G12V*^*/Rabex-5*^*DPYT*^*; c765gal4/+* (Fig 3E’, S4E’ Fig)

*w; UAS Ras*^*Y4F*,*G12V*^*/+; c765gal4/+* (Fig 3C, 3F and 3I, S4C Fig, S4F Fig, S4I Fig)

*w; UAS Ras*^*Y4F*,*G12V*^*/Rabex-5*^*DPYT*^*; c765-gal4/+* (Fig 3F’, S4F’ Fig)

*w; ey-gal4/+* (Fig 4A, left eye in 4D and 4F, S5A Fig, left eye in S5D Fig, S5F Fig)

*w; ey-gal4/UAS Ras*^*G12V*^ (Fig 4B, right eye in 4D and 4E, S5B Fig, right eye in S5D Fig, S5E Fig)

*w; ey-gal4/UAS Ras*^*Y4E*,*G12V*^ (Fig 4C, left in in 4E, right eye in 4F, S5C Fig, left eye in S5E Fig, right eye in S5F Fig)

*w; GMR-gal4/+* (Fig 4G, Fig 5C and 5C’, S1H–S1H” Fig, S5G Fig, S7A Fig)

*w; GMR-gal4/UAS Ras*^*G12V*^ (Fig 4H, Fig 5D and 5D’, S5H Fig, right eye in S5J Fig, S7B Fig)

*w; GMRgal4/ UAS Ras*^*Y4E*,*G12V*^ (Fig 4I, S5I Fig, left eye in S5J Fig)

*w; He-gal4*, *UAS GFP*.*nls/+* (Fig 4J, S5K Fig)

*w; UAS Ras*^*G12V*^*/+UAS GFP*.*nls/+* (Fig 4K, S5L Fig)

*w; UAS Ras*^*Y4E*,*G12V*^*/+; UAS GFP*.*nls/+* (Fig 4L, S5M Fig)

*MS1096-gal4; UAS Ras*^*Y4E*,*G12V*^ (Fig 4Q, S5R Fig)

*w; UAS Ras*^*Y4E*,*G12V*^*/+; c765-gal4/+* (Fig 4O and 4U, S5P Fig, S5V Fig)

*w; UAS Ras*^*Y4E*,*G12V*^*; c765-gal4* (Fig 4S, S5T Fig)

*w; UAS Rabex-5*^*IR*^*/+; c765-gal4/+* (Fig 4T, S5U Fig)

*w; UAS Rabex-5*^*IR*^*/ UAS Ras*^*Y4E*,*G12V*^*; c765gal4/+* (Fig 4V, S5W Fig)

*w; GMR-gal4/+; UAS hop*^*IR*^*/+* (Fig 5F and 5F’)

*w; GMR-gal4/UAS Ras*^*G12V*^*; UAS hop*^*IR*^*/+* (Fig 5G and 5G’)

*w; GMR-gal4/UAS Ras*^*Y4F*,*G12V*^*; UAS hop*^*IR*^*/+* (Fig 5H and 5H’)

*w; GMR-gal4/+; UAS Rabex-5*^*DPYT*^*/+* (S1I–S1I” Fig)

*w; GMR-gal4/UAS Ras*^*Y4F*,*G12V*^ (Fig 5E and 5E’, S7C Fig)

*w; GMR-gal4/UAS Ras*^*G12V*^*; UAS EGFR/+* (S7C Fig)

*w; GMR-gal4/UAS Ras*^*G12V*^*; UAS EGFR* (S7C Fig)

Genotypes of flies in Western blots:

*w; UAS Ras*^*WT*^*/+; Act5C-gal4/+* (S3B Fig lane 1)

*w; UAS Ras*^*Y4E*^*/+; Act5C-gal4/+* (S3B Fig lane 2)

*w; UAS Ras*^*Y4F*^*/+; Act5C-gal4/+* (S3B Fig lane 3)

### Statistical analysis

Wings were measured using Image J software. Wing size comparisons were analyzed using GraphPad Prism software. Unpaired T-tests were used to compare two groups (e.g. controls versus Ras^Y4F^ over-expressing wings), and one way ANOVA analysis was used for experiments considering three groups (e.g. control versus Ras^WT^ versus Ras^Y4F^). Chi square analysis (using the CHITEST function in Microsoft Excel was used to compare percentages in Fig 1E–1E” for percent of Ras constructs conjugated to ubiquitin and in Fig 5I to compare the percentage of eyes with black tissue. The expected values for each comparison were calculated based on control values applied to the N for other samples.

## Supporting information

S1 Fig An N-terminal Tyrosine-based signal directs Ras for mono- and di-ubiquitination. (A) *Drosophila* Ras and H-, N-, and K-Ras share significant homology in their N-termini but differ substantially in a C-terminal region called the HyperVariable Region (HVR). The HVR differs between H-, N, and K-Ras and directs the specific localization of each isoform [19–21]. Schematic showing a series of deletions tested for ubiquitination of *Drosophila* Ras in S2 cells indicating which deletion constructs supported Ras mono- and di-ubiquitination in S2 cells. Deletion constructs were tagged with FLAG, His6, and GFP at the N-terminus (not depicted). To ensure that C-terminal deletions maintained appropriate localization, all constructs deleting the C-terminus maintained the C-terminal CAAX localization signal, CKML (shown in red). * indicates that this construct in some experiments showed poly-ubiquitination (sample gel shown in D). (B-D) Sample gels corresponding to many of the constructs in the schematic in (A). Specific constructs are indicated above the gel according to the abbreviations listed in (A) for constructs A-K. (B-B’) N- and C-terminal deletions show ubiquitination pattern of full length Ras for only the N-terminal construct. (B) Un-adjusted gels. (B’) Gels from (B) were adjusted to highlight the mono- and di-ubiquitination pattern (or lack thereof); brightness and contrast adjustments were applied to the entire images. (C-C’) 20 amino acid constructs in the N-terminal 80 amino acids for low levels of expression (C) and in over-loaded conditions (C’). Only the N-terminal 20 amino acids (construct J) consistently shows ubiquitin conjugates. Mono- and di-ubiquitin conjugates are never seen for 21–40 and 41–60, and never predominate for 61–80 even for high levels of expression (C’). (D) More than once, we saw poly-ubiquitin conjugates for the tagged 61–80 region (construct G), shown here in comparison to 1–60 which gives the standard Ras pattern of predominantly mono- and di-ubiquitin conjugates. This was seen multiple times, but was not consistent. This may mean that a degradation signal for Nedd4, βTRCP, and LZTR1 could lie in this region, or this could be an artefact of exposing a cryptic degron. We have not resolved this as this was outside the scope of this work. We include this here for transparency. (E) DNA encoding competitive peptides of 1-20CKML or 61-80CKML tagged with MYC and GFP were transfected at the same levels (1X) as Ras^WT^ or in five-fold excess (5X). Consistently, over-expression of the 1-20CKML peptide but not the 61-80CKML peptide inhibited formation of Ras^WT^ ubiquitin conjugates. (F) Another example highlights the consistency of ubiquitination of 1–10 and 1–20 but not 61–80. (G) Larger gel of cropped images from Fig 1A. The bands recognized by both anti-FLAG and anti-HA antibodies represent ubiquitinated species of Ras (marked by an asterisk, *). Other bands in the anti-HA gel reflect non-Ras, co-purifying ubiquitinated proteins. (H-I”) Pupal eyes dissected 48 hours after puparium formation were stained with antibodies to E-cadherin (Ecad, blue in H-H’ and I-I’; DSHB, catalog # DCAD2, rat monoclonal primary antibodies; goat anti-Rat Alexa Fluor 647 Invitrogen, Catalog # A21247 secondary antibodies) and anti-Pan Ras antibodies to recognize endogenous Ras (red in H, H”, I, I”; Millipore Sigma, catalog # OP40100UG, mouse monoclonal primary antibodies; goat anti-mouse Alexa Fluor 555 Invitrogen, Catalog #A21422 secondary antibodies). (H-H”) Staining of control GMR-gal4/+ pupal eyes shows the pattern of Ecad (blue) and endogenous Ras (red) in the pupal eye. Merge shown in H. (I-I”) Staining of Rabex-5^DPYT^ expressing pupal eyes shows redistribution of Ras (red) to an internal compartment. Merge in I. Boxes in H-I” represent 50 micron areas.
(TIF)

S2 Fig Ras Tyrosine 4 is important for Ras ubiquitination. (A-A’) Sample gels showing ubiquitin conjugates of alanine substitution mutants. (A) Gel showing M1A, T2A, E3A, Y4A, K5A, L6A, and V7A mutants compared to control transfected cells (lane 1) and control Ras^WT^ (lane 2). Reproducibly, we see decreased ubiquitination for Ras^Y4A^ and Ras^V7A^ mutants (red boxes). We see no decrease or no reproducible decrease for other alanine substitution mutants. (A’) Gel showing E3A, Y4A, K5A, L6A, V7A, V8A, and V9A in the Ras^G12V^ context compared to control Ras^WT^ (lane 2) and control Ras^G12V^ (lane 3) or control-transfected cells (lane 1). Typically, Ras^G12V^ shows greater ubiquitin conjugation than Ras^WT^ (lane 3 compared to lane 2). This gel is at saturation for Ras^WT^, therefore this may be an underestimate of the increased ubiquitination of Ras^G12V^. (B) Schematic summarizing the results of alanine scanning of the first 10 amino acids of Ras (in the context of full length Ras^WT^ or Ras^G12V^) highlighting substitution mutants for which we saw decreased ubiquitination reproducibly. Alanine substitution of Y4 and V7 in otherwise wild-type Ras^WT^ (which is primarily in the GDP-loaded conformation) reproducibly decreased ubiquitination. Alanine substitution at E3, Y4, K5, and V7 in Ras^G12V^ shows decreased ubiquitination compared to Ras^G12V^ (which is in the GTP-loaded conformation). We could not address the role of G10; Ras^G10A^ and Ras^G10A,G12V^ mutants mislocalized within the cell (H for Ras^G10A^ below). (C) Alignment showing complete conservation of the N-terminal 10 amino acids of *Drosophila* Ras and human H-Ras, N-Ras, and K-Ras. Alignment also shows conservation of the tyrosine and valine in *Drosophila Ras2*. (D) Gel showing Rabex-5 mediated increase in ubiquitin conjugates for *Drosophila* Ras^WT^ and also for *Drosophila Ras2*. (E-M) FLAG-His6 tagged Ras alanine mutants that decreased ubiquitination showed localization to the membrane and association in intracellular puncta as did FLAG-His6 Ras^WT^ and FLAG-His6 Ras^G12V^ controls suggesting that the decreased ubiquitination did not result from inappropriate localization. (E-E”) Typically, Ras is seen at the membrane and at intracellular puncta. Staining a population of cells transfected with Ras^WT^ (green, reflecting FLAG staining) reveals individual cells with more membrane-associated Ras (E), Ras at the membrane and in intracellular puncta (E’) or Ras enriched in intracellular puncta (E”). Populations of cells (>100 per sample) were scored blind by multiple lab members to ensure appropriate localization was confirmed. Ras localization is shown for (E-E”) Ras^WT^, (F) Ras^Y4A^, (G) Ras^V7A^, (H) Ras^G10A^, (I) Ras^G12V^, (J) Ras^E3A,G12V^, (K) Ras^Y4A,G12V^, (L) Ras^K5A,G12V^, and (M) Ras^V7A,G12V^. Ras^G10A^ mutants mislocalized (H) so their ubiquitination could not be appropriately interpreted. Ras^Y4A^, Ras^V7A^,. Ras^E3A,G12V^, Ras^Y4A,G12V^, Ras^K5A,G12V^, and Ras^V7A,G12V^ mutants show localization to the membrane and intracellular puncta where Ras^WT^ and Ras^G12V^ localize, so the decreased ubiquitination did not result from a failure to localize to these compartments in the cell. Boxes in E-M represent 20 μm square regions. (N) S2 cells were transfected with empty vector or FLAG-His6 tagged Ras. Nickel pulldowns from cell lysates showed no recognition by anti-phospho-tyrosine (anti-ptyr) antibodies or anti-FLAG antibodies for vector-control lysates (lane 1) but showed a signal for Ras^WT^ lysates confirming that *Drosophila* Ras is tyrosine phosphorylated. (O-P) FLAG-His6 Ras purified from S2 cells using nickel beads is recognized by anti-ptyr antibodies (lane 1). Phenylalanine substitution mutant Ras^Y4F^ reproducibly shows decreased recognition (lane 2). Total Ras is shown by anti-FLAG antibodies. Quantification is done by normalizing for the amount of Ras pulled down. Quantification of a sample gel a the upper end of the range (O) shows decreased recognition of Ras^Y4F^ compared to Ras^WT^ by approximately 27% and a sample gel at the lower end of the rage (P) shows a decrease by 17%. Gels at either end of the range are shown for transparency. Typically, we see Ras isolated from cells run as a doublet (obvious in O) although this can be less obvious depending on the separation on the gel.
(TIF)

S3 Fig Non-phosphorylatable Ras shows Ras gain-of-function phenotypes *in vivo*. (A) Summary table of various gal4 drivers used to express Ras^WT^. In many cases, Ras^WT^ expression does not cause an obvious, visible phenotype. (B) Expressing FLAG-His6 tagged Ras^WT^, Ras^Y4E^, or Ras^Y4F^ transgenes using *Act5C-gal4* results in similar expression levels. Western blot shows anti-FLAG (the tag on Ras transgenes) and anti-tubulin loading control. (C) Control wing (*Tub-gal4/+*). (D) Ras^WT^ expression driven by *Tub-gal4*. Ectopic longitudinal vein material is seen anterior to the L2 longitudinal vein (arrow, enlarged in D’) and on the posterior crossvein (arrow). (E) Ras^Y4F^ expression driven by *Tub-gal4*. Ectopic longitudinal vein material is seen anterior and posterior to the L2 longitudinal vein (arrow, enlarged in E’). The ectopic wing vein phenotype (arrows) is enhanced upon Y4F mutation (compare E’ to D’). (F) Control homozygous *MS1096-gal4* wing. (G) Wing homozygous for *MS1096-gal4* and *UAS Ras*^*WT*^. Extra wing vein material is obvious, particularly where the longitudinal veins meet the wing margin (arrows). (H) Wing homozygous for *MS1096-gal4* and *UAS Ras*^*Y4F*^. The extra wing vein phenotype (arrows) is enhanced compared to Ras^WT^. Male wings are shown in C-H. (I) Summary table of transgene rescue experiments. Expressing FLAG-His6 tagged Ras^WT^, Ras^Y4E^, or Ras^Y4F^ transgenes using *Act5C-gal4* rescues the early lethality of Ras^e1b^/Ras^e1b^; flies survive to the pupal stage.
(TIF)

S4 Fig Non-phosphorylatable oncogenic Ras enhances oncogenic Ras phenotypes). (A) Control wing (*c765-gal4/+*) at 18°C. (B) Ras^G12V^ expressed using *c765-gal4* at 18°C causes subtle wing effects. (C) Ras^Y4F,G12V^ shows an increase in wing vein effects. (D) Control wing (*c765-gal4/+*) at 21°C. (D’) Control wing expressing low level of Rabex-5^DPYT^ using *c765-gal4* at 21°C causes no wing vein disruption. (E) Ras^G12V^ expressed using *c765-gal4* at 21°C causes extra wing veins and thickened veins. (E’) Rabex-5^DPYT^ expression concurrent to Ras^G12V^ using *c765-gal4* at 21°C suppresses the extra wing veins and thickened vein phenotypes. (F) Ras^Y4F,G12V^ expressed using *c765-gal4* at 21°C shows an increase in wing effects including reduction in size compared to Ras^G12V^. (F’) Rabex-5^DPYT^ expression concurrent to Ras^Y4F,G12V^ using *c765-gal4* at 21°C shows a similar phenotype as Ras^Y4F,G12V^. (G) Control wing (*c765-gal4/+*) at 22°C. (H) Ras^G12V^ expressed using *c765-gal4* at 22°C causes a more severe phenotype than at 21°C. (I) Ras^Y4F,G12V^ expressed using *c765-gal4* at 22°C shows further wing disruption compared to Ras^G12V^. Male wings are shown.
(TIF)

S5 Fig Ras Y4 phosphomimic suppresses the phenotypes of oncogenic Ras dependent on the presence of Rabex-5. (A-F) Y4E phosphomimic mutation suppresses the eye overgrowth and outgrowth phenotypes of Ras^G12V^. Control eye (*ey-gal4/+*) (A, left eye in D, left eye in F). Oncogenic Ras, Ras^G12V^, driven by *ey-gal4* (B, right eye in D and E). Ras^Y4E,G12V^ driven by *ey-gal4* (C, left eye in E, right eye in F). Head-to-head photos in D-F highlight the suppression of overgrowth. (G) Control *GMR-gal4/+* eye. (H) Oncogenic Ras, Ras^G12V^, driven by *GMR-gal4*. (I) Ras^Y4E,G12V^ driven by *GMR-gal4*. Y4E phosphomimic mutation suppresses phenotypes of Ras^G12V^. (J) Overhead shot showing a Ras^Y4E,G12V^ driven by *GMR-gal4* (left head) and a Ras^G12V^ driven by *GMR-gal4* (right head). Arrows indicate small spots of black tissue in the Ras^G12V^ eyes that are absent in the morphologically normal Ras^Y4E,G12V^ eyes. Male eyes are shown in A-J. (K-M) *He-gal4* was used to drive Ras transgene expression in hemocytes. To visualize hemocytes, a *UAS GFP* transgene was also used. The entire larvae from images in Fig 4J–4L are shown. With this driver, there is strong background fluorescence in the salivary glands in the anterior region of the larva (cropped out of the panel in the main figure). (K) Control, GFP driven by *He-gal4*. (L) Ras^G12V^ and GFP driven by *He-gal4*. (N) Ras^Y4E,G12V^ and GFP driven by *He-gal4*. Larvae in K-M were imaged at the same settings. Tracings of larvae in K and M indicate larval outlines. Excess hemocytes are evident in (L) by the strong GFP signal (green). The excess hemocyte phenotype is suppressed upon Y4E mutation. Scale bars in K-M indicate 1.5 mm. (N) Control wing *(c765-gal4/+)*. (O) Oncogenic Ras, Ras^G12V^, driven by *c765-gal4*. (P) Ras^Y4E,G12V^ driven by *c765gal4*. Y4E phosphomimic mutation suppresses the extra wing vein phenotype of Ras^G12V^. (Q) Control homozygous *MS1096-gal4* wing. (R) Wing homozygous for *MS1096-gal4* and Ras^Y4E,G12V^. Oncogenic Ras driven by *MS1096-gal4* is lethal; Y4E phosphomimic mutation yields obvious wing phenotypes but suppresses the lethality of one copy or two copies of Ras^G12V^. (S) Control *c765-gal4/+* wing. (T) Wing homozygous for *c765-gal4* and Ras^Y4E,G12V^ transgene shows the obvious extra wing vein phenotype associated with oncogenic Ras. (U) Low-level Rabex-5 RNAi driven by *c765-gal4* yields no visible phenotype. (V) Ras^Y4E,G12V^ expression driven by *c765-gal4* shows very subtle or no extra wing vein phenotypes. (W) Low-level Rabex-5 RNAi elicits obvious extra wing vein phenotypes (arrows) of Ras^Y4E,G12V^ expression driven by *c765-gal4*. Male wings are shown in N-W.
(TIF)

S6 Fig JAK2, SRC, and EGFR promote Ras phosphorylation at Y4. (A) Gel of Schneider S2 cell extracts probed with anti-pY4 antibodies. The peptide polyclonal anti-Y4 antibodies recognize many bands in non-transfected control cells (“empty,” lane 2), in Ras^WT^-transfected cells (lane 3), and in Ras^G12V^-tranfected cells (lane 4). Molecular weight markers (lane 1) indicate 15, 25, 35, and 55 kDa protein sizes; given the recognition of so many bands by the anti-pY4 antibodies, it is impossible to distinguish over-expressed Ras proteins from other cross-reacting proteins from cell extracts. (B) Recombinant Ras^WT^ protein purified from bacteria was incubated in the presence (lane 2) or absence (lane 1) of 100 micrograms of recombinant JAK2. Despite low-level recognition of unmodified Ras^WT^ protein in lane 1, there is an obvious dramatic increase in recognition of Ras^WT^ protein when incubated with JAK2 in lane 2 (anti-pY4 antibodies, upper gel; anti-Pan Ras antibodies, lower gel). Because the recognition was so dramatic, we were concerned that any cross-reaction with JAK2 itself could not be distinguished from Ras. Therefore, we titrated the amount of JAK2 in kinases assays and could reliably see a response using 20 micrograms of kinase used in subsequent assays. Using a lower level of kinase allowed us to distinguish modified Ras from cross-reacting JAK2 and SRC shown in subsequent panels. (C-D) Full gel of gel slices shown in Fig 5A. (C) Ras^WT^ protein incubated in the presence or absence of JAK2, EGFR, or SRC proteins. Increased recognition of Ras^WT^ protein by anti-pY4 antibodies is seen upon incubation with JAK2 (lane 2) and SRC (lane 4) but not EGFR (lane 3) compared to unmodified protein (lane 1). (D) Ras^G12V^ protein incubated in the presence or absence of JAK2, EGFR, or SRC proteins. Increased recognition of Ras^G12V^ protein by anti-pY4 antibodies is seen upon incubation with JAK2 (lane 2), EGFR (lane 3) and SRC (lane 4) compared to unmodified protein (lane 1). Ras^Y4F^ protein incubated in the presence or absence of JAK2, EGFR, or SRC proteins. No difference in recognition of Ras^Y4F^ protein by anti-pY4 antibodies is seen upon incubation with JAK2 (lane 2), EGFR (lane 3) and SRC (lane 4) compared to unmodified protein (lane 1). Basal recognition of Ras^Y4F^ protein by anti-pY4 antibodies is decreased compared to Ras^WT^ protein. (E-H) Full gels of gel slices from Fig 5B. (E) Ras^WT^ protein incubated in the presence or absence of JAK2, EGFR, or SRC proteins. Increased recognition of Ras^WT^ protein by anti-pY4 antibodies is seen upon incubation with JAK2 (lane 5) and SRC (lane 7) but not EGFR (lane 6) compared to unmodified protein (lane 1). (F) Ras^G12V^ protein incubated in the presence or absence of JAK2, EGFR, or SRC proteins. Increased recognition of Ras^G12V^ protein by anti-pY4 antibodies is seen upon incubation with JAK2 (lane 5) and SRC (lane 7) but not this preparation of EGFR (lane 6) compared to unmodified protein (lane 1). (G) Ras^Y4F^ protein incubated in the presence or absence of JAK2, EGFR, or SRC proteins. No difference in recognition of Ras^Y4F^ protein by anti-pY4 antibodies is seen upon incubation with JAK2 (lane 5), EGFR (lane 6) and SRC (lane 7) compared to unmodified protein (lane 1). (H) Ras^Y4F,G12V^ protein incubated in the presence or absence of JAK2, EGFR, or SRC proteins. No difference in recognition of Ras^Y4F^ protein by anti-pY4 antibodies is seen upon incubation with JAK2 (lane 5), EGFR (lane 6) and SRC (lane 7) compared to unmodified protein (lane 1). Anti-pY4 antibodies also recognize JAK2 (indicated by “J” in C-H) and SRC (indicated by “S” in C-H), but this recognition does not interfere with detection of Ras proteins that run at a different size.
(TIF)

S7 Fig (A) Control *GMR-gal4/+* eye. (B) Ras G12V expressed using *GMR-gal4*. Eyes are rough and show some loss of eye pigment. Some eyes have black tissue at the periphery of the eye (arrow). (C) Ras^Y4F,G12V^ expressed using *GMR-gal4*. Eyes are rough and show some loss of eye pigment. Some eyes have black tissue at the periphery of the eye (arrow). Eyes in A-C also appear in Fig 5, as these experiments were done concurrently. (D) EGFR driven by *GMR-gal4* control eye. Eyes are rough and show some loss of eye pigment. (E) EGFR expressed concurrently with Ras^G12V^ using *GMR-gal4*. Eyes are rough, show more dramatic loss of eye pigment throughout the eye, and show consistent black tissue in the anterior periphery of the eye (arrow). (F) EGFR expressed concurrently with Ras^Y4F,G12V^ using *GMR-gal4*. Eyes are rough, show more dramatic loss of eye pigment throughout the eye, and show consistent black tissue in the anterior periphery of the eye (arrow). Eyes also consistently show black tissue in other regions of the eye (arrowhead). Female eyes are shown.
(TIF)

S1 File Raw Data for Fig 1E–1E”. Data tables indicating (1) the raw measurements from Image J for mono- and di-ubiquitin conjugates or unconjugated (full length) protein for RasWT, Rabex-5+RasWT, RasY4E, RasY4E, Rabex-5+RasY4E, RasY4F, and Rabex-5+RasY4F, (2) the corresponding percentage calculations graphed in Fig 1E’, and (3) calculations of the relative Rabex-5 mediated ubiquitination (normalizing the calculations to the percent ubiquitination of Rabex-5+RasWT) graphed in Fig 1E”.
(XLSX)

S2 File Raw data for Fig 2G. Raw measurements (pixel counts of wing tracings) for homozygous *ms1096-gal4* control wings, wings homozygous for *ms1096-gal4* and *UAS RasWT*, and wings homozygous for *ms1096-gal4* and *UAS RasY4F*. Calculations of the average area for each set of wings are shown. Also shown are calculations to give the normalized wing areas (raw wing size divided by the average wing area for ms1096-gal4 control wings), and averages of normalized wing areas graphed in Fig 2G.
(XLSX)

S3 File Raw data for Fig 5I. Raw data reporting the number of eyes with no black tissue or eyes with black tissue for eyes in which *GMR-gal4* is driving expression of RasG12V, RasG12V concurrent to *hop* RNAi, RasY4F,G12V, and RasY4F,G12V concurrent to *hop* RNA. The recorded data and percentage calculations are shown.
(XLSX)

S1 Table K-Ras, N-Ras, and H- Ras ubiquitination sites. Table summarizing reported sites of lysine (K) ubiquitination of mouse and human K-Ras, N-Ras, and H-Ras, their reported biological roles, and their conservation in *Drosophila* Ras.
(PDF)
