# Supplementary figures and images for: A conserved, N-terminal tyrosine signal directs Ras for inhibition by Rabex-5
Source: PLoS Genet. 2020 Jun 19;16(6):e1008715. doi: 10.1371/journal.pgen.1008715 (PMC7329146; doi:10.1371/journal.pgen.1008715)

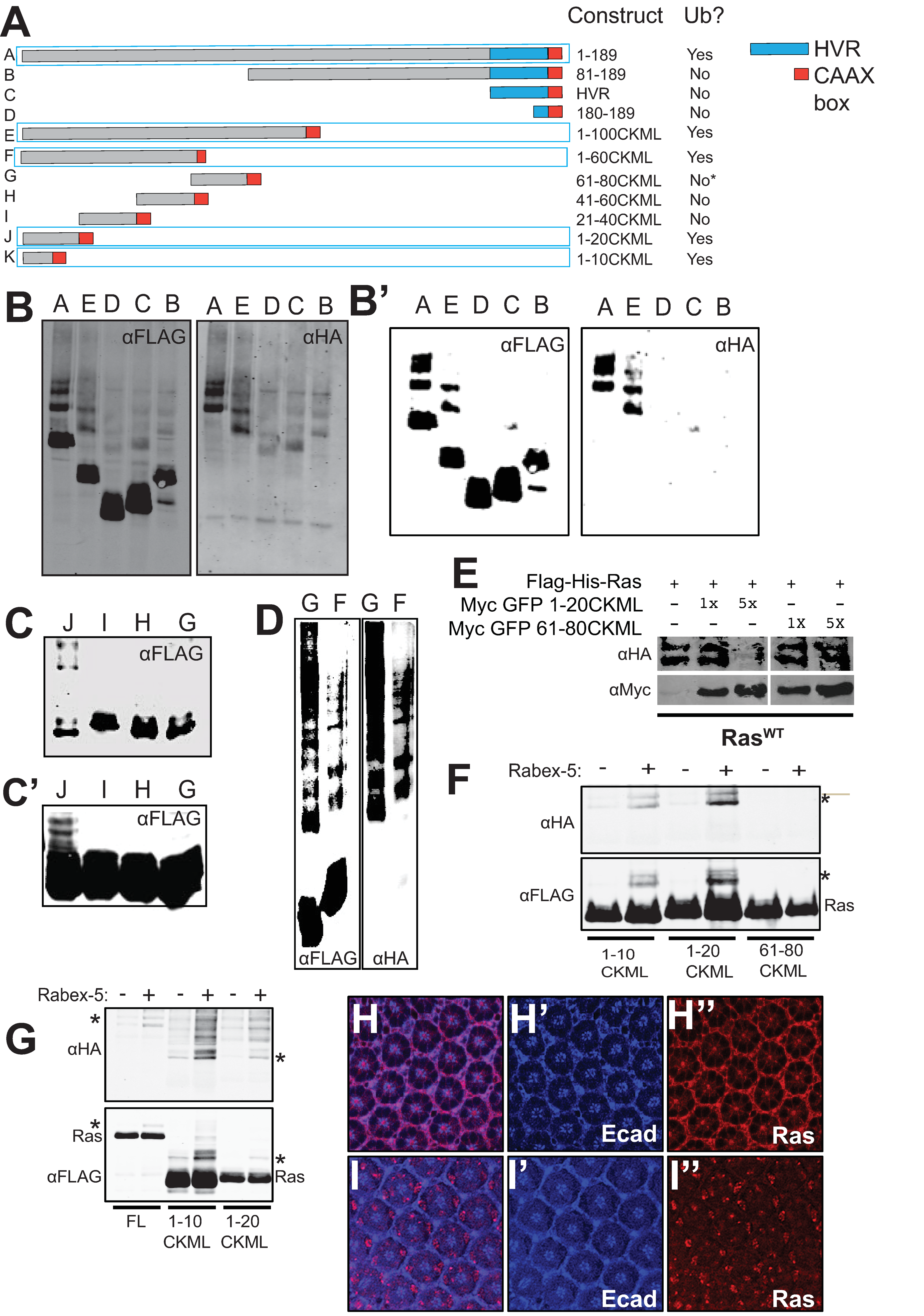

Supplement: S1 Fig — (A) Drosophila Ras and H-, N-, and K-Ras share significant homology in their N-termini but differ substantially in a C-terminal region called the HyperVariable Region (HVR). The HVR differs between H-, N, and K-Ras and directs the specific localization of each isoform [19–21]. Schematic showing a series of deletions tested for ubiquitination of Drosophila Ras in S2 cells indicating which deletion constructs supported Ras mono- and di-ubiquitination in S2 cells. Deletion constructs were tagged with FLAG, His6, and GFP at the N-terminus (not depicted). To ensure that C-terminal deletions maintained appropriate localization, all constructs deleting the C-terminus maintained the C-terminal CAAX localization signal, CKML (shown in red). * indicates that this construct in some experiments showed poly-ubiquitination (sample gel shown in D). (B-D) Sample gels corresponding to many of the constructs in the schematic in (A). Specific constructs are indicated above the gel according to the abbreviations listed in (A) for constructs A-K. (B-B’) N- and C-terminal deletions show ubiquitination pattern of full length Ras for only the N-terminal construct. (B) Un-adjusted gels. (B’) Gels from (B) were adjusted to highlight the mono- and di-ubiquitination pattern (or lack thereof); brightness and contrast adjustments were applied to the entire images. (C-C’) 20 amino acid constructs in the N-terminal 80 amino acids for low levels of expression (C) and in over-loaded conditions (C’). Only the N-terminal 20 amino acids (construct J) consistently shows ubiquitin conjugates. Mono- and di-ubiquitin conjugates are never seen for 21–40 and 41–60, and never predominate for 61–80 even for high levels of expression (C’). (D) More than once, we saw poly-ubiquitin conjugates for the tagged 61–80 region (construct G), shown here in comparison to 1–60 which gives the standard Ras pattern of predominantly mono- and di-ubiquitin conjugates. This was seen multiple times, but was not consistent. This [file pgen.1008715.s001.tif]

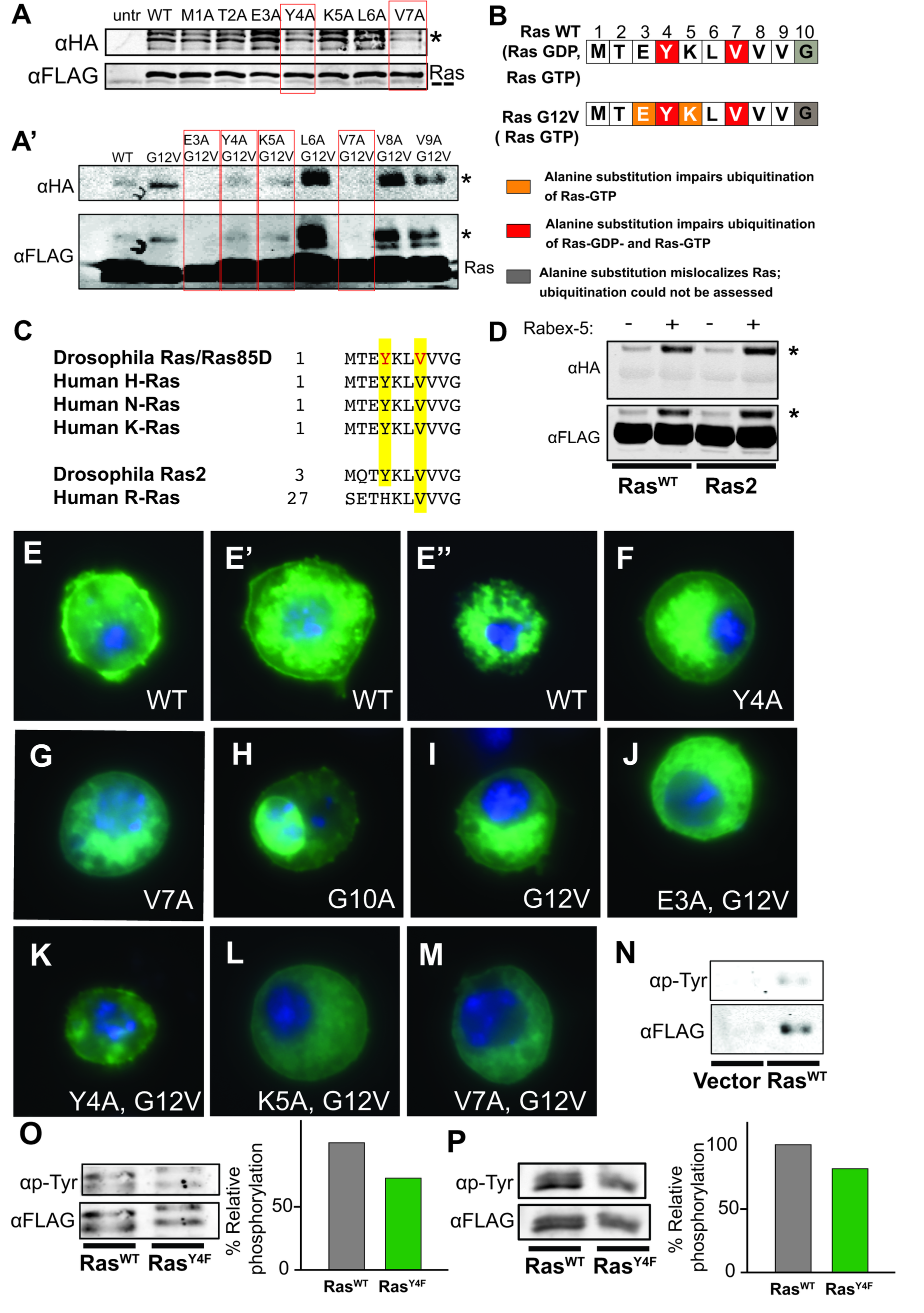

Supplement: S2 Fig — (A-A’) Sample gels showing ubiquitin conjugates of alanine substitution mutants. (A) Gel showing M1A, T2A, E3A, Y4A, K5A, L6A, and V7A mutants compared to control transfected cells (lane 1) and control RasWT (lane 2). Reproducibly, we see decreased ubiquitination for RasY4A and RasV7A mutants (red boxes). We see no decrease or no reproducible decrease for other alanine substitution mutants. (A’) Gel showing E3A, Y4A, K5A, L6A, V7A, V8A, and V9A in the RasG12V context compared to control RasWT (lane 2) and control RasG12V (lane 3) or control-transfected cells (lane 1). Typically, RasG12V shows greater ubiquitin conjugation than RasWT (lane 3 compared to lane 2). This gel is at saturation for RasWT, therefore this may be an underestimate of the increased ubiquitination of RasG12V. (B) Schematic summarizing the results of alanine scanning of the first 10 amino acids of Ras (in the context of full length RasWT or RasG12V) highlighting substitution mutants for which we saw decreased ubiquitination reproducibly. Alanine substitution of Y4 and V7 in otherwise wild-type RasWT (which is primarily in the GDP-loaded conformation) reproducibly decreased ubiquitination. Alanine substitution at E3, Y4, K5, and V7 in RasG12V shows decreased ubiquitination compared to RasG12V (which is in the GTP-loaded conformation). We could not address the role of G10; RasG10A and RasG10A,G12V mutants mislocalized within the cell (H for RasG10A below). (C) Alignment showing complete conservation of the N-terminal 10 amino acids of Drosophila Ras and human H-Ras, N-Ras, and K-Ras. Alignment also shows conservation of the tyrosine and valine in Drosophila Ras2. (D) Gel showing Rabex-5 mediated increase in ubiquitin conjugates for Drosophila RasWT and also for Drosophila Ras2. (E-M) FLAG-His6 tagged Ras alanine mutants that decreased ubiquitination showed localization to the membrane and association in intracellular puncta as did FLAG-His6 RasWT and FLAG-His6 RasG12V controls suggesting that the de [file pgen.1008715.s002.tif]

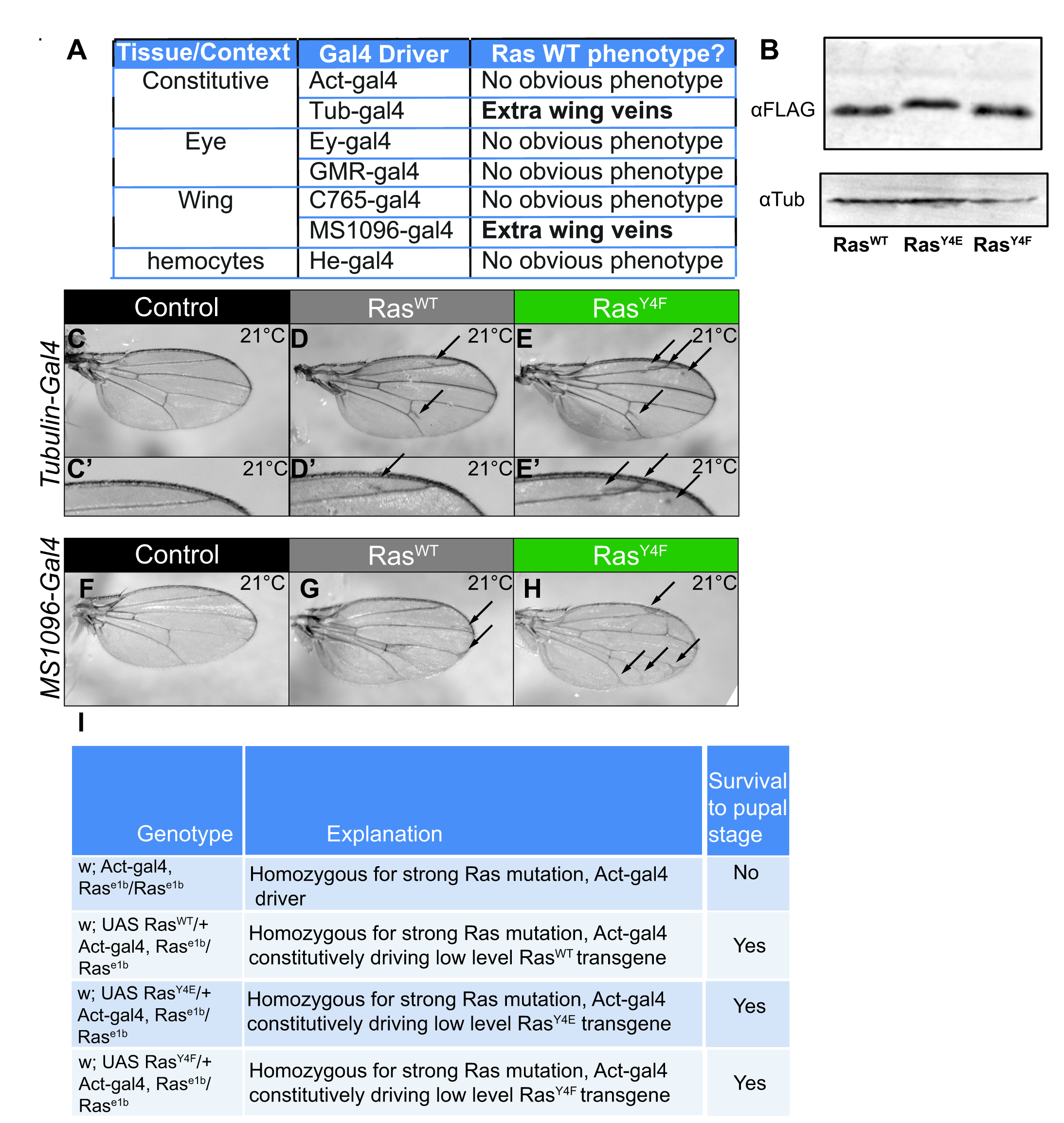

Supplement: S3 Fig — (A) Summary table of various gal4 drivers used to express RasWT. In many cases, RasWT expression does not cause an obvious, visible phenotype. (B) Expressing FLAG-His6 tagged RasWT, RasY4E, or RasY4F transgenes using Act5C-gal4 results in similar expression levels. Western blot shows anti-FLAG (the tag on Ras transgenes) and anti-tubulin loading control. (C) Control wing (Tub-gal4/+). (D) RasWT expression driven by Tub-gal4. Ectopic longitudinal vein material is seen anterior to the L2 longitudinal vein (arrow, enlarged in D’) and on the posterior crossvein (arrow). (E) RasY4F expression driven by Tub-gal4. Ectopic longitudinal vein material is seen anterior and posterior to the L2 longitudinal vein (arrow, enlarged in E’). The ectopic wing vein phenotype (arrows) is enhanced upon Y4F mutation (compare E’ to D’). (F) Control homozygous MS1096-gal4 wing. (G) Wing homozygous for MS1096-gal4 and UAS RasWT. Extra wing vein material is obvious, particularly where the longitudinal veins meet the wing margin (arrows). (H) Wing homozygous for MS1096-gal4 and UAS RasY4F. The extra wing vein phenotype (arrows) is enhanced compared to RasWT. Male wings are shown in C-H. (I) Summary table of transgene rescue experiments. Expressing FLAG-His6 tagged RasWT, RasY4E, or RasY4F transgenes using Act5C-gal4 rescues the early lethality of Rase1b/Rase1b; flies survive to the pupal stage. (TIF) [file pgen.1008715.s003.tif]

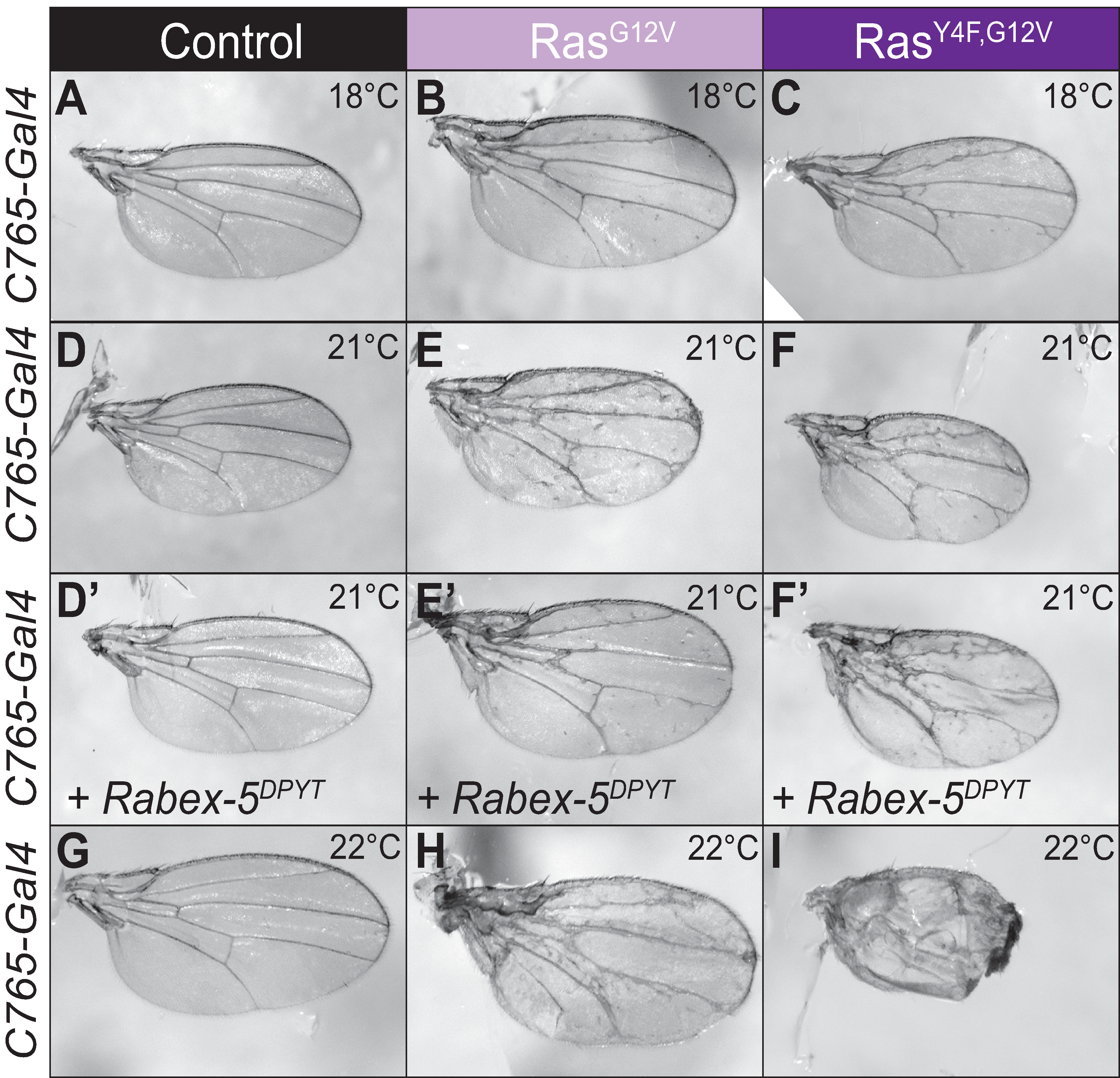

Supplement: S4 Fig — (A) Control wing (c765-gal4/+) at 18°C. (B) RasG12V expressed using c765-gal4 at 18°C causes subtle wing effects. (C) RasY4F,G12V shows an increase in wing vein effects. (D) Control wing (c765-gal4/+) at 21°C. (D’) Control wing expressing low level of Rabex-5DPYT using c765-gal4 at 21°C causes no wing vein disruption. (E) RasG12V expressed using c765-gal4 at 21°C causes extra wing veins and thickened veins. (E’) Rabex-5DPYT expression concurrent to RasG12V using c765-gal4 at 21°C suppresses the extra wing veins and thickened vein phenotypes. (F) RasY4F,G12V expressed using c765-gal4 at 21°C shows an increase in wing effects including reduction in size compared to RasG12V. (F’) Rabex-5DPYT expression concurrent to RasY4F,G12V using c765-gal4 at 21°C shows a similar phenotype as RasY4F,G12V. (G) Control wing (c765-gal4/+) at 22°C. (H) RasG12V expressed using c765-gal4 at 22°C causes a more severe phenotype than at 21°C. (I) RasY4F,G12V expressed using c765-gal4 at 22°C shows further wing disruption compared to RasG12V. Male wings are shown. (TIF) [file pgen.1008715.s004.tif]

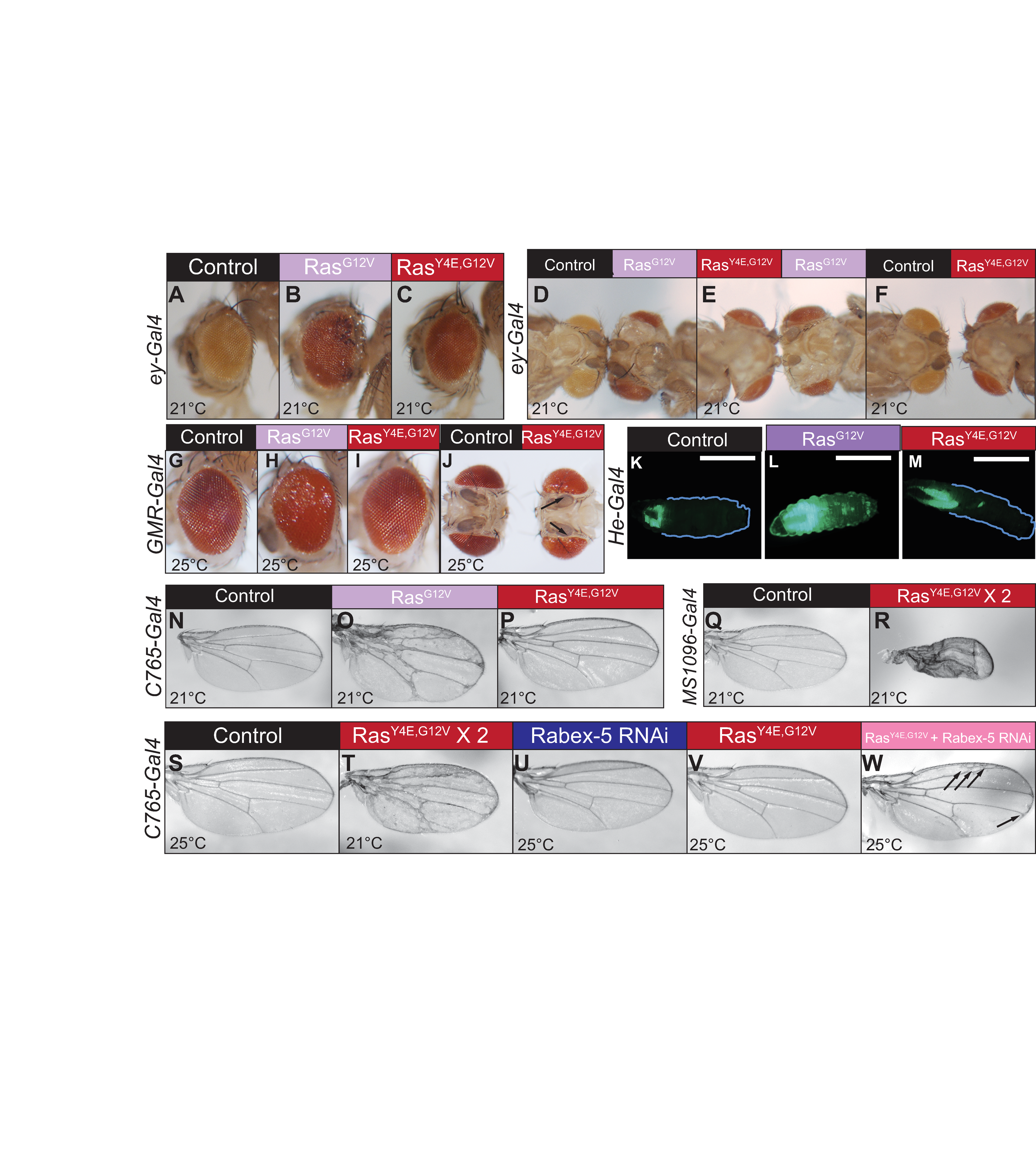

Supplement: S5 Fig — (A-F) Y4E phosphomimic mutation suppresses the eye overgrowth and outgrowth phenotypes of RasG12V. Control eye (ey-gal4/+) (A, left eye in D, left eye in F). Oncogenic Ras, RasG12V, driven by ey-gal4 (B, right eye in D and E). RasY4E,G12V driven by ey-gal4 (C, left eye in E, right eye in F). Head-to-head photos in D-F highlight the suppression of overgrowth. (G) Control GMR-gal4/+ eye. (H) Oncogenic Ras, RasG12V, driven by GMR-gal4. (I) RasY4E,G12V driven by GMR-gal4. Y4E phosphomimic mutation suppresses phenotypes of RasG12V. (J) Overhead shot showing a RasY4E,G12V driven by GMR-gal4 (left head) and a RasG12V driven by GMR-gal4 (right head). Arrows indicate small spots of black tissue in the RasG12V eyes that are absent in the morphologically normal RasY4E,G12V eyes. Male eyes are shown in A-J. (K-M) He-gal4 was used to drive Ras transgene expression in hemocytes. To visualize hemocytes, a UAS GFP transgene was also used. The entire larvae from images in Fig 4J–4L are shown. With this driver, there is strong background fluorescence in the salivary glands in the anterior region of the larva (cropped out of the panel in the main figure). (K) Control, GFP driven by He-gal4. (L) RasG12V and GFP driven by He-gal4. (N) RasY4E,G12V and GFP driven by He-gal4. Larvae in K-M were imaged at the same settings. Tracings of larvae in K and M indicate larval outlines. Excess hemocytes are evident in (L) by the strong GFP signal (green). The excess hemocyte phenotype is suppressed upon Y4E mutation. Scale bars in K-M indicate 1.5 mm. (N) Control wing (c765-gal4/+). (O) Oncogenic Ras, RasG12V, driven by c765-gal4. (P) RasY4E,G12V driven by c765gal4. Y4E phosphomimic mutation suppresses the extra wing vein phenotype of RasG12V. (Q) Control homozygous MS1096-gal4 wing. (R) Wing homozygous for MS1096-gal4 and RasY4E,G12V. Oncogenic Ras driven by MS1096-gal4 is lethal; Y4E phosphomimic mutation yields obvious wing phenotypes but suppresses the lethality of one copy or two copies of Ras [file pgen.1008715.s005.tif]

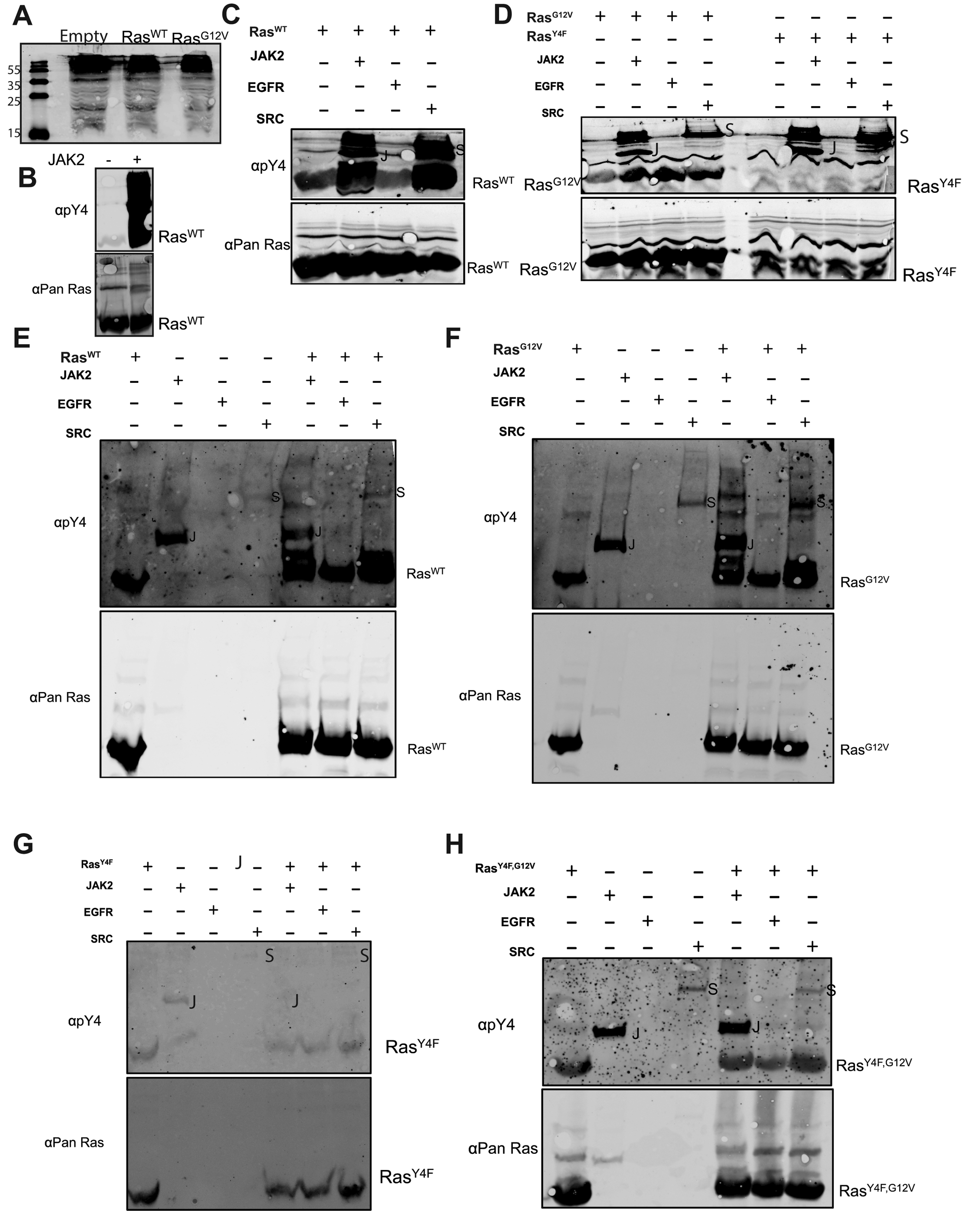

Supplement: S6 Fig — (A) Gel of Schneider S2 cell extracts probed with anti-pY4 antibodies. The peptide polyclonal anti-Y4 antibodies recognize many bands in non-transfected control cells (“empty,” lane 2), in RasWT-transfected cells (lane 3), and in RasG12V-tranfected cells (lane 4). Molecular weight markers (lane 1) indicate 15, 25, 35, and 55 kDa protein sizes; given the recognition of so many bands by the anti-pY4 antibodies, it is impossible to distinguish over-expressed Ras proteins from other cross-reacting proteins from cell extracts. (B) Recombinant RasWT protein purified from bacteria was incubated in the presence (lane 2) or absence (lane 1) of 100 micrograms of recombinant JAK2. Despite low-level recognition of unmodified RasWT protein in lane 1, there is an obvious dramatic increase in recognition of RasWT protein when incubated with JAK2 in lane 2 (anti-pY4 antibodies, upper gel; anti-Pan Ras antibodies, lower gel). Because the recognition was so dramatic, we were concerned that any cross-reaction with JAK2 itself could not be distinguished from Ras. Therefore, we titrated the amount of JAK2 in kinases assays and could reliably see a response using 20 micrograms of kinase used in subsequent assays. Using a lower level of kinase allowed us to distinguish modified Ras from cross-reacting JAK2 and SRC shown in subsequent panels. (C-D) Full gel of gel slices shown in Fig 5A. (C) RasWT protein incubated in the presence or absence of JAK2, EGFR, or SRC proteins. Increased recognition of RasWT protein by anti-pY4 antibodies is seen upon incubation with JAK2 (lane 2) and SRC (lane 4) but not EGFR (lane 3) compared to unmodified protein (lane 1). (D) RasG12V protein incubated in the presence or absence of JAK2, EGFR, or SRC proteins. Increased recognition of RasG12V protein by anti-pY4 antibodies is seen upon incubation with JAK2 (lane 2), EGFR (lane 3) and SRC (lane 4) compared to unmodified protein (lane 1). RasY4F protein incubated in the presence or absence of JAK2, EGFR, or SR [file pgen.1008715.s006.tif]

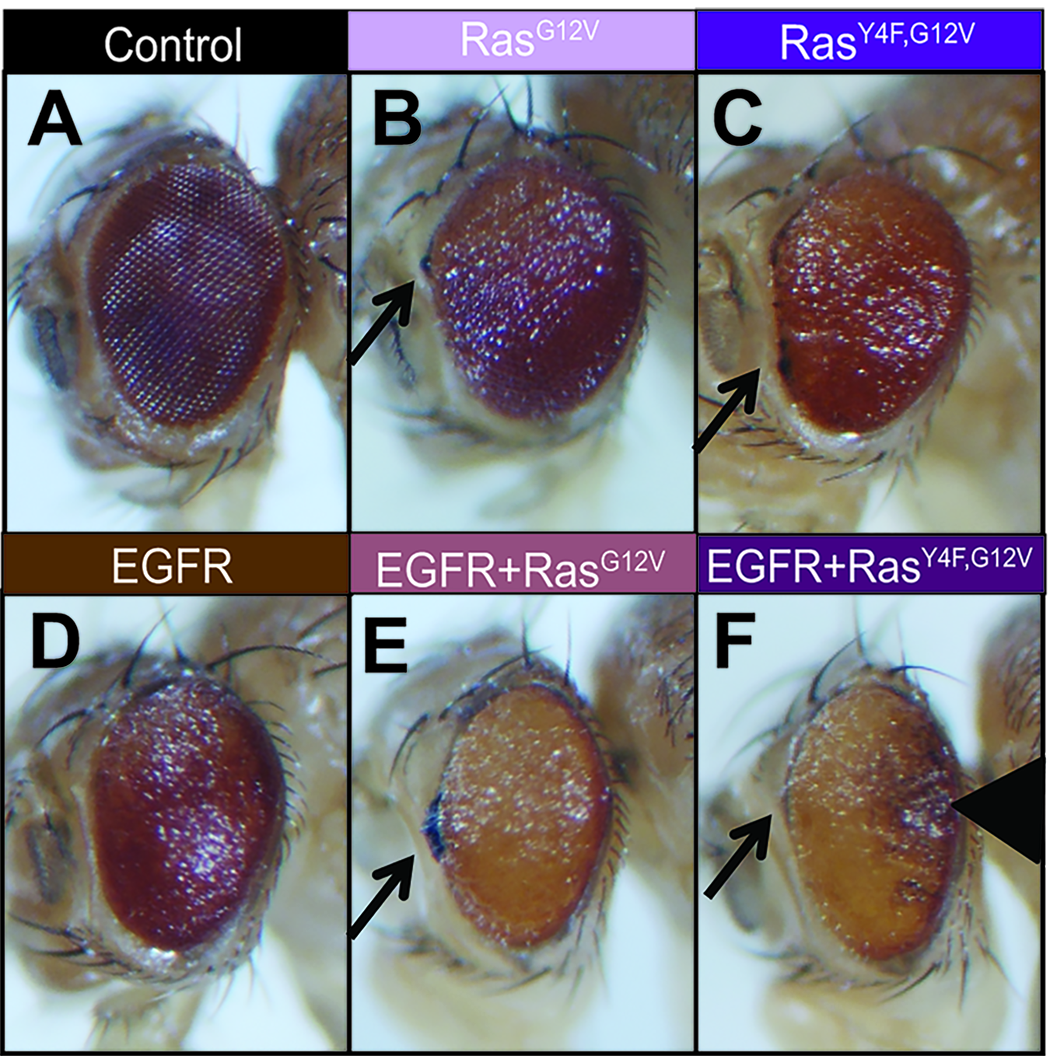

Supplement: S7 Fig — (A) Control GMR-gal4/+ eye. (B) Ras G12V expressed using GMR-gal4. Eyes are rough and show some loss of eye pigment. Some eyes have black tissue at the periphery of the eye (arrow). (C) RasY4F,G12V expressed using GMR-gal4. Eyes are rough and show some loss of eye pigment. Some eyes have black tissue at the periphery of the eye (arrow). Eyes in A-C also appear in Fig 5, as these experiments were done concurrently. (D) EGFR driven by GMR-gal4 control eye. Eyes are rough and show some loss of eye pigment. (E) EGFR expressed concurrently with RasG12V using GMR-gal4. Eyes are rough, show more dramatic loss of eye pigment throughout the eye, and show consistent black tissue in the anterior periphery of the eye (arrow). (F) EGFR expressed concurrently with RasY4F,G12V using GMR-gal4. Eyes are rough, show more dramatic loss of eye pigment throughout the eye, and show consistent black tissue in the anterior periphery of the eye (arrow). Eyes also consistently show black tissue in other regions of the eye (arrowhead). Female eyes are shown. (TIF) [file pgen.1008715.s007.tif]
