# Supplementary material for: A conserved, N-terminal tyrosine signal directs Ras for inhibition by Rabex-5
Source: PLoS Genet. 2020 Jun 19;16(6):e1008715. doi: 10.1371/journal.pgen.1008715 (PMC7329146; doi:10.1371/journal.pgen.1008715)
Supplement: S1 Table — Table summarizing reported sites of lysine (K) ubiquitination of mouse and human K-Ras, N-Ras, and H-Ras, their reported biological roles, and their conservation in Drosophila Ras. (PDF) [file pgen.1008715.s011.pdf]

K	Mouse (K, N, or H)	Human (K, N, or H)	Role of ubiquitin?	Conserved in *Drosophila?*
**K101**	H-Ras [37]		unreported	Yes
**K104**		K-Ras [38-39]	Unreported; no effect on activity in *in vitro* assays [39]	Yes
**K117**	K-Ras [37]	K-Ras [37, 40-41] N-Ras [41], H-Ras [38]	Activation [38-39] (increases nucleotide dissociation/GDP-GTP-exchange [39])	Yes
**K128**	K-Ras [37], N-Ras [37]	K-Ras [40-42] N-Ras [40-41, 43-44]	unreported	No
**K147**	K-Ras [37], N-Ras [37], H-Ras [37]	K-Ras [38], N-Ras 41,43], H-Ras [38-39, 41, 43, 45]	Activation [38-39] (Impaired GTP-hydrolysis by GAPs [39])	Yes
**K170**	H-Ras [37, 20]	H-Ras [38]	Inhibition; placed by LZTR1 [20]	There is a K170 (and other nearby lysines) in the fly HVR; given the sequence variation, it is unclear if a specific lysine is conserved

S1 Table: K-Ras, N-Ras, and H- Ras ubiquitination sites. Table summarizing reported sites of lysine (K) ubiquitination of mouse and human K-Ras, N-Ras, and H-Ras, their reported biological roles, and their conservation in *Drosophila* Ras.
